# Temperature Compensation of Embedded Fiber Bragg Gratings in Deep-Sea Syntactic Foam Error Bounds and Strain Identifiability

**DOI:** 10.3390/s26206483

**Published:** 2026-10-13

**Authors:** Kun Wang, Qinbo Zhang, Xie Zhao, Wei Zhang

**Affiliations:** 1 China Ship Scientific Research Center, Wuxi 214082, Chinazhaoxie@cssrc.com.cn (X.Z.); 2 Key Laboratory of Ship Structure Safety, Wuxi 214082, China; 3 Technical Institute of Physics and Chemistry, Chinese Academy of Sciences, Beijing 100190, China; heyz@cssrc.com.cn

**Keywords:** fiber Bragg grating, syntactic foam, deep-sea monitoring, temperature compensation, strain transfer, identifiability

## Abstract

Fiber Bragg gratings (FBGs) embedded in deep-sea syntactic foam are attractive for in situ structural monitoring, but their wavelength response conflates mechanical strain, temperature, and hydrostatic pressure, and the foam remains thermally unequilibrated during deployment. This study develops a quantitative framework for separating these contributions and for allocating the errors that limit mechanical strain interpretation. We report sea-trial measurements from a specimen instrumented with two strain-sensing channels (S1, S2) and two temperature-reference channels (T1, T2), deployed to approximately 4.6 km depth in the Philippine Sea. A two-channel observation model links finite-grating strain transfer, reference-channel mechanical leakage, and spatial temperature mismatch to the compensation error, and is complemented by a coupled thermomechanical–optical validation against the field data. During descent the optical strain indication reached approximately −4.4 × 10^3^ με, and after a four-hour station keeping hold the internal temperature still exceeded the 1.6 °C seawater by 7.7 °C, confirming that thermal transients dominate the residual signal. Nominal thermal correction accounted for 76.7% of the channel-summed station keeping increment and 88.0% of the deck recovery increment—single-deployment, channel-summed point estimates rather than independent replicates. For a 10 με error allocation, the temperature mismatch limit is 0.228 °C under ideal transfer and isolation, tightening to 0.164 °C at a transfer efficiency of 0.8 and weighted leakage of 0.10, and to 0.127 °C with an additional 20 με spatial strain mismatch. The framework converts these observations into quantitative requirements for sensor pairing, calibration stability, and mechanical strain interpretation, and shows that independent thermal information improves mechanical-amplitude identifiability even when the augmented condition number increases.

## 1. Introduction

Syntactic foams provide buoyancy under external pressure through hollow glass microspheres dispersed in a polymer matrix. Microsphere properties, matrix stiffness, and interface integrity govern their deformation and failure [1,2,3]. Microsphere damage can also increase water uptake and reduce buoyancy [4]. Embedded fiber Bragg gratings (FBGs) offer access to local deformation during deployment, but their wavelength shifts combine mechanical, thermal, and direct pressure responses. Separating these contributions is essential when the foam remains out of thermal equilibrium during descent or station keeping.

Tian et al. combined femtosecond-inscribed FBG arrays with different temperature coefficients to monitor buoyancy materials [5]. They subsequently resolved temperature and strain gradients during curing at submillimeter spacing [6]. In the cured material, the fiber–host interface controls mechanical strain transfer [7], while microsphere content modifies elastic and viscoelastic behavior [8]. These dependencies raise a deployment-specific question: how accurately must paired gratings share temperature and host strain for their signals to support a mechanical strain estimate?

Classical FBG theory [9] and dual-parameter sensing [10,11] provide the sensitivity-matrix framework for strain–temperature separation. Interface mechanics and shear-lag models explain deviations between bare-fiber and embedded sensitivities [12,13,14], and direct optical pressure response introduces an additional term [15]. The material itself can deform through several mechanisms [16,17]. Consequently, the measurement problem involves both inversion of optical sensitivities and identification of material-response parameters. These operations have different requirements for independent information [18,19,20].

Bulk hydrostatic measurements [21] and in situ tomography [22] constrain material deformation and microstructural change at scales complementary to FBG measurements. Reinforcement also changes the mechanical and thermal behavior of syntactic foam [23]. Studies of curing [24,25], polymer-fiber embedding [26], thermal residual stress [27], and composite dual-parameter sensors [28] establish the importance of host–sensor coupling. A deployment model must retain this coupling while distinguishing the calibrated optical indication from the mechanical quantity sought.

Here, sea-trial stage increments quantify the contribution that nominal temperature correction makes to the optical strain indication. A coupled observation model then converts transfer loss, reference-channel leakage, and spatial mismatch into a joint sensor-pairing criterion. The analysis also links calibration covariance to strain uncertainty and separates temporal amplitude identifiability from sensitivity-matrix invertibility. The contribution is a quantitative error allocation for embedded, nonisothermal measurements, built on established optical discrimination and strain transfer theory.

Dual-parameter composite sensors [28], compact dual gratings [29], and FBG–multimode-interference systems [30] discriminate strain and temperature through differing sensitivities; the present analysis extends that inverse framework to the mismatch and transfer conditions of an embedded, nonisothermal deployment, and its practical output is a sensor-pairing requirement that tightens as mechanical leakage and spatial strain mismatch increase. Published measurements show that microsphere content changes viscoelastic behavior [8], while microsphere buckling and interface debonding contribute to foam failure [16]; matrix modification [17] and reinforcement [23] also change the material response; and bulk hydrostatic measurements [21] and in situ tomography [22] constrain deformation at complementary scales. These mechanisms provide context for the delayed optical evolution reported below, and their contributions enter the observation model through distinct quantities.

Deep-sea syntactic-foam monitoring imposes specific metrological requirements that frame the analysis below. Buoyancy modules for the 3000–6000 m depth class operate at 30–60 MPa hydrostatic pressure, with seawater temperature decreasing from roughly 28 °C at the surface to 1.5–2 °C at depth. An embedded sensor must therefore resolve axial strains of several thousand microstrain (the present specimen reached about −4400 με at 4.6 km depth) together with internal temperatures spanning 0–30 °C. For buoyancy and structural-health accounting, the quantities of practical interest are the long-term axial strain at the 10–20 με level and the internal temperature at the 0.1–0.5 °C level. Given the measured temperature-error gain of 43–44 με/°C, a 10 με design allocation corresponds to approximately 0.2 °C of temperature mismatch (Section 4). These ranges and accuracies are the benchmark against which the reported 76.7%/88.0% thermal fractions and the residual tens of microstrain must be judged.

## 2. Materials and Methods

### 2.1. Specimen and Sensor Arrangement

**Figure 1:**
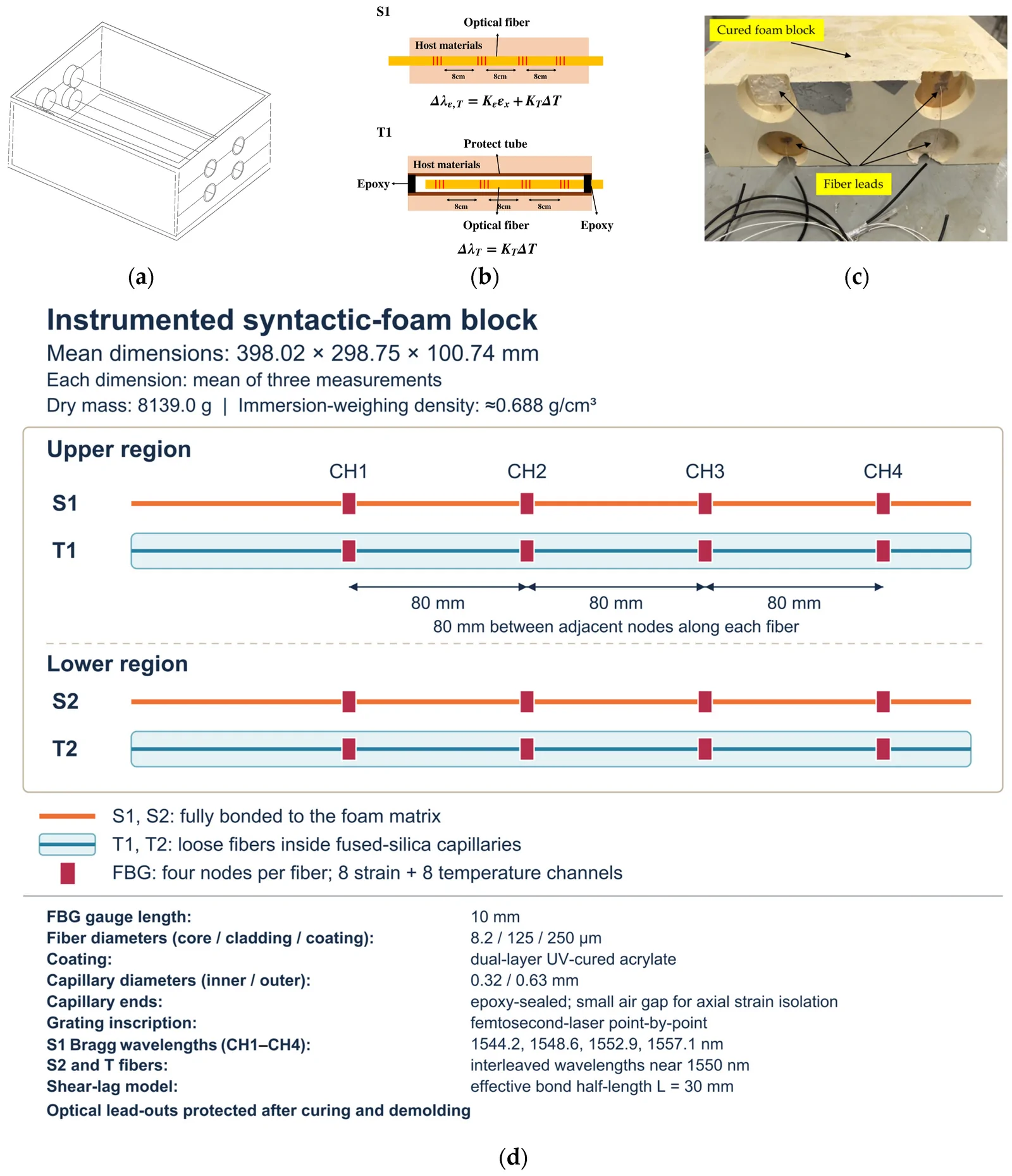
Sensor arrangement and syntactic foam specimen. (**a**) Specimen geometry; (**b**) bonded strain fibers and capillary-isolated temperature fibers; (**c**) cured specimen and optical leads; (**d**) schematic of the sensor arrangement.

The instrumented syntactic foam specimen contained strain-sensing fibers S1 and S2 in the upper and lower regions, alongside temperature-sensing fibers T1 and T2. Each fiber contained four FBGs at 80 mm intervals, giving eight strain channels and eight temperature channels. The strain fibers were directly coupled to the matrix, while loose capillary tubes reduced axial strain transfer to the temperature fibers. After curing and demolding, the optical lead-out regions were protected for deployment. The dry mass was 8139.0 g, and the mean dimensions were 398.02 × 298.75 × 100.74 mm. The separately reported immersion-weighing density was approximately 0.688 g/cm^3^. Each specimen dimension was measured three times and averaged. Figure 1 shows the sensor arrangement and specimen, and the layout of fiber Bragg grating nodes.

The strain-sensing and temperature-reference gratings were inscribed by femtosecond-laser point-by-point writing in single-mode fiber (SMF-28e+, Corning Incorporated, Corning, NY, USA) with a core diameter of 8.2 μm, a cladding diameter of 125 μm, and a dual-layer UV-cured acrylate coating of 250 μm outer diameter. The gratings had a gauge length of 10 mm, and adjacent gratings along a fiber were spaced 80 mm apart, giving four channels (CH1–CH4) per fiber; the channels CH1–CH4 therefore denote the four grating nodes distributed along each fiber (Figure 1a). The nominal Bragg wavelengths of the four S1 channels were 1544.2, 1548.6, 1552.9, and 1557.1 nm, with S2 and the T fibers occupying interleaved wavelengths near 1550 nm to avoid spectral overlap. The strain fibers were bonded over their full embedded length, whereas the temperature fibers were threaded through fused-silica capillaries (inner diameter 0.32 mm, outer diameter 0.63 mm, Polymicro Technologies, Molex LLC, Phoenix, AZ, USA) whose ends were sealed with epoxy, leaving a small air gap so that axial strain transfer to the grating was largely decoupled while thermal contact was retained. The shear-lag model of Section 2.5 uses an effective bond half-length *L* = 30 mm.

The mean external dimensions and the dry mass give a geometric density of 8139.0 g/(39.802 × 29.875 × 10.074 cm^3^) = 0.679 g/cm^3^, whereas immersion (Archimedes) weighing gives 0.688 g/cm^3^. The 1.3% difference is attributed to the rounded edges and chamfered corners of the molded specimen, which make the rectangular envelope slightly larger than the actual displaced volume, and to a thin surface film retained during dimensional measurement; the immersion value therefore approximates the true envelope-excluded density. The mass was measured to ±0.1 g (balance resolution 0.01 g) and each dimension to ±0.02 mm, giving a geometric-density uncertainty of approximately ±0.001 g/cm^3^, and the immersion density is reported to ±0.002 g/cm^3^ from repeated weighing and the water-density correction.

### 2.2. Pressure Exposure and Sea-Trial Measurements

**Figure 2:**
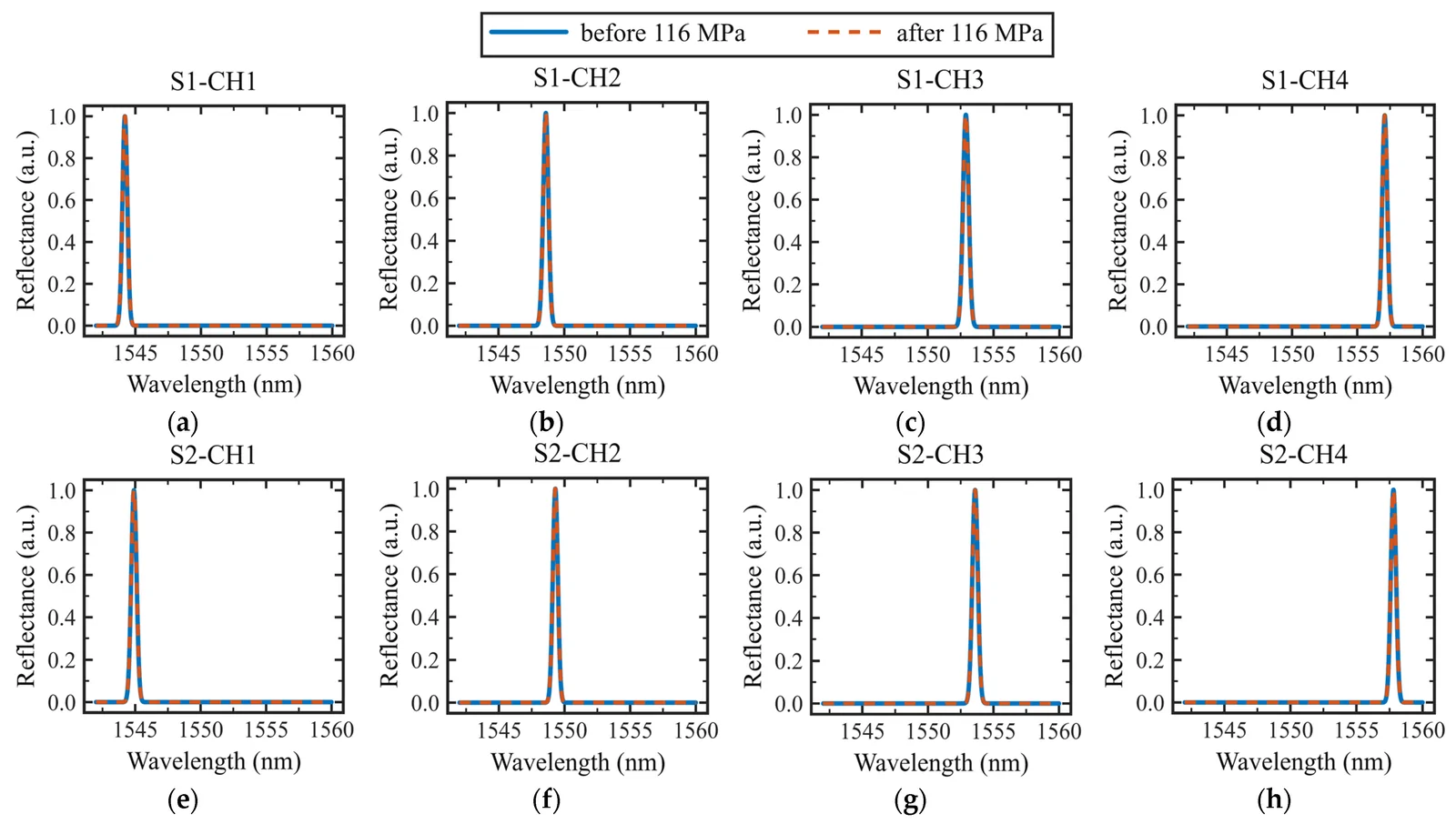
Reflection spectra of the eight strain gratings before and after hydrostatic exposure at 116 MPa. (**a**–**d**) S1-CH1–CH4; (**e**–**h**) S2-CH1–CH4.

Across eight gratings, the measured bandwidths were 0.44–0.51 nm before exposure and 0.45–0.50 nm afterward; the largest absolute change was 0.02 nm. The principal peaks remained distinguishable, enabling subsequent wavelength interrogation. Spectral interpretation depends on grating structure [31], nonuniform and residual thermal strain [27], and the peak-tracking method [32]. This comparison evaluates peak preservation; the compensation analysis below uses the separately reported calibration coefficients. The channels CH1–CH4 denote the four gratings distributed along each strain fiber; the eight strain gratings comprise CH1–CH4 on S1 and CH1–CH4 on S2; and the eight individual spectra are shown in Figure 2.

Separate laboratory water-uptake tests used two specimens. A 116 MPa hold for 24 h increased the mass from 8137.7 to 8142.5 g, corresponding to 0.059% uptake. A smaller specimen subjected to 100 pressure cycles to 116 MPa increased from 1410.5 to 1412.5 g, corresponding to 0.142% uptake. These mass changes characterize the specified laboratory exposures and are distinct from the sea-trial optical measurements. Specimens were conditioned by drying at 60 °C under vacuum until the mass change was below 0.01 g over 24 h and then cooled to (23 ± 2) °C in a desiccator; the elapsed time between decompression and weighing was kept below 30 min. Weighing used an analytical balance (Mettler Toledo, Greifensee, Switzerland) with 0.01 g resolution, and the combined conditioning and surface-drying reproducibility gives a mass uncertainty of ±0.05 g (i.e., ±0.0006% for the larger specimen). These mass gains provide an independent lower-bound estimate of the buoyancy loss that must be separated from the optical thermal correction, and they bound the density drift that affects the strain–modulus link and the zero-crossing interpretation during deck recovery.

**Figure 3:**
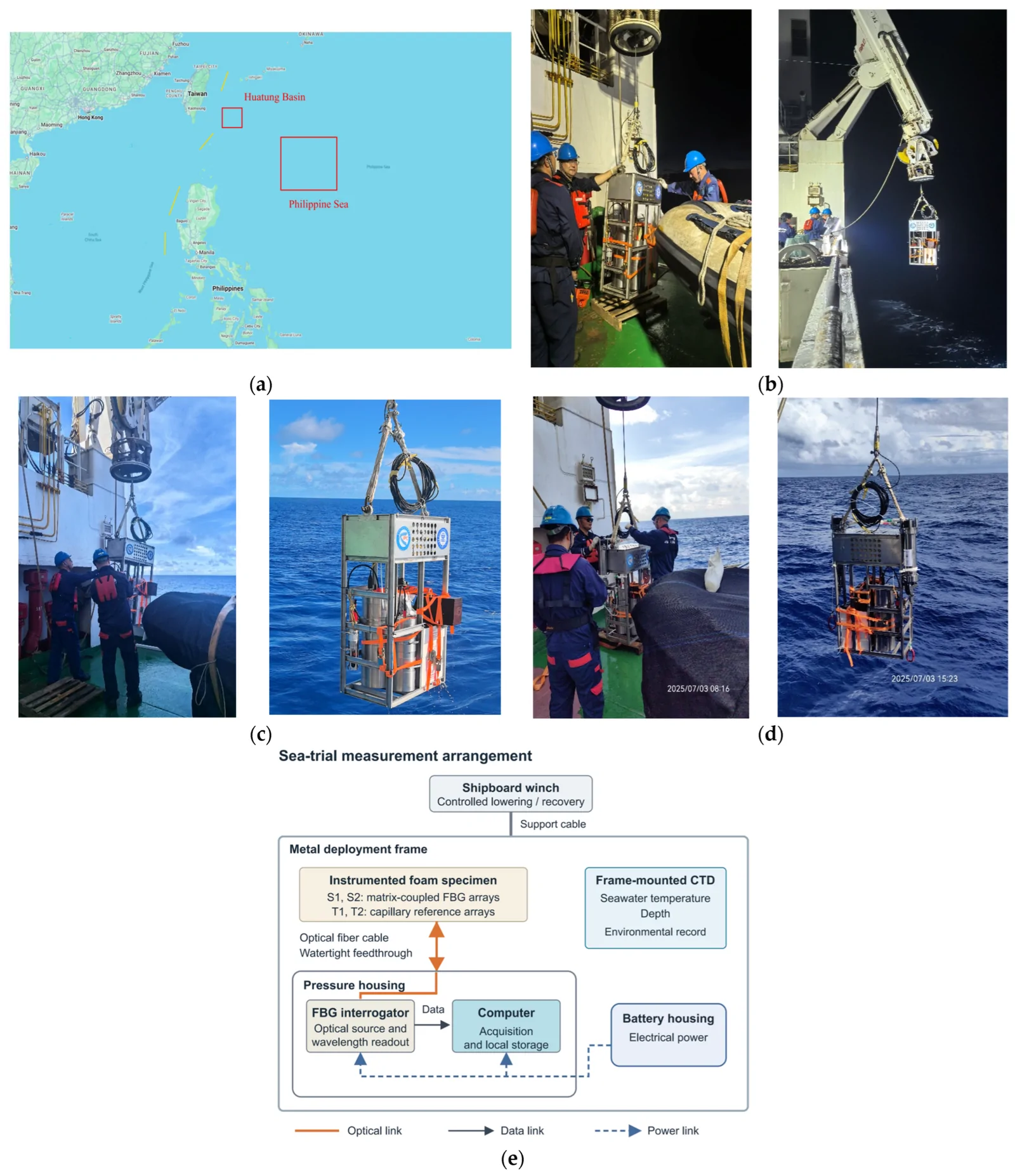
Sea-trial region and deployment operations. (**a**) Regional map showing the Huatung Basin (upper red box) and Philippine Sea (lower red box); (**b**) on-site photos of the first deployment and recovery; (**c**) on-site photos of the second deployment and recovery; (**d**) on-site photos of the third deployment and recovery; (**e**) technical schematic of the frame-mounted specimen, optical interrogation and power/data paths. Non-Latin labels denote place names.

The specimen was secured in a metal frame and connected through watertight cabling to a pressure housing containing the interrogator, computer, and batteries. A frame-mounted conductivity–temperature–depth (CTD) instrument (SBE 49 FastCAT, Sea-Bird Scientific, Bellevue, WA, USA) recorded depth and seawater temperature. The winch was operated at a nominal deployment and recovery speed of 55 m/min, with a target station keeping duration of at least 4 h. During cruise TS-47-2 aboard the research vessel (R/V) Tansuo-1 in the Philippine Sea, the same specimen reached 4587, 4637, and 4588 m on three deployments in June–July 2025. Figure 3 shows the regional setting and deployment operations. A software fault interrupted recording during the first deployment, and water ingress contaminated T2 with pressure response during the second. Quantitative stage correction uses the valid S1/T1 pairs from the second deployment; Table 1 identifies the use of each record. The FBG interrogator provided a wavelength resolution of 1 pm, a repeatability of 0.3 pm, and a sampling rate of 1 Hz over the 1520–1570 nm range; a 10 min wavelength standard deviation of 0.8523 pm (0.687 με) confirmed that the instrument resolution and stability are sufficient to resolve the 0.07–0.9 pm/°C between-channel slope differences reported in Section 3.1. A technical diagram of the complete setup is shown in Figure 3e.

The three deployments provide repeated observations of one instrumented specimen. All 16 gratings remained optically interrogable before and after each deployment. The specimen remained mounted between deployments and showed no visible surface cracking when removed after the third deployment. Optical continuity and measurement validity were assessed separately, as illustrated by the compromised T2 temperature response.

The field optical strain indication is denoted by $\varepsilon_{\mathrm{eq}}=\left(\Delta \lambda_{S}-\Delta \lambda_{T}\right)/K_{\varepsilon }$, with $K_{\varepsilon }=1.2405$ pm/με as reported for the acquisition conversion. At equal channel temperatures, wavelength subtraction retains a temperature gain $g=\left(b_{S}-b_{T}\right)/K_{\varepsilon }$. For a stage increment, $r=\delta \varepsilon_{\mathrm{eq}}-g\delta T_{\mathrm{stage}}$, where δ denotes end minus start and $\delta T_{\mathrm{stage}}$ is the T1 temperature increment. Table 2 and Table 3 use this coefficient; the analytical scenarios use its rounded value of 1.24 pm/με. Section 3.2 relates *r* to the mechanical and pressure contributions; Section 4 treats departures from equal channel temperature.

### 2.3. Temperature Calibration and Statistical Treatment

Table 2 summarizes the eight strain-channel temperature slopes, their channel statistics, and the paired S1/T1 temperature sensitivities and compensation coefficients. The mean, sample standard deviation, and coefficient of variation describe between-channel dispersion. The channel statistics characterize spatial consistency within the specimen; they are not replicate calibration uncertainties.

An ordinary least-squares fit to eleven displacement-stage (Newport Corporation, Irvine, CA, USA) calibration points over 0–10,000 με yielded a sensitivity of 1.2405 pm/με ($R^{2}=0.9999867$). The S1/T1 temperature calibration comprised five temperature levels over approximately 6–36 °C. The linear fits gave R^2^ = 0.9991–0.9996 for S1 and 0.9978–0.9998 for T1. The marginal standard uncertainties of the fitted slopes were 0.060 pm/°C for S1/S2 and 0.020 pm/°C for T1/T2. Propagating these, together with *u*(*K_ε_*), through *g* = (*b*_S_ − *b*_T_)/*K_ε_* gives *u*(*g*) ≈ 0.05 με/°C, the gain uncertainty used in Table 3. T1 slopes for CH1–CH4 were 14.06, 13.85, 13.79, and 13.97 pm/°C, respectively. Combining them with the S1 slopes gives equal-subtraction gains of 31.94–32.65 με/°C (Table 2). The nominal calibration slopes are $b_{S}$ and $b_{T}$. The coefficient ratio is *c* = *b_S_*/*b_T_*, and *g* = (*b_S_* − *b_T_*)/*K*_ε_ uses *K*_ε_ = 1.2405 pm/με. Data fitting and post-processing were performed using MATLAB R2023a (The MathWorks, Natick, MA, USA).

**Figure 4:**
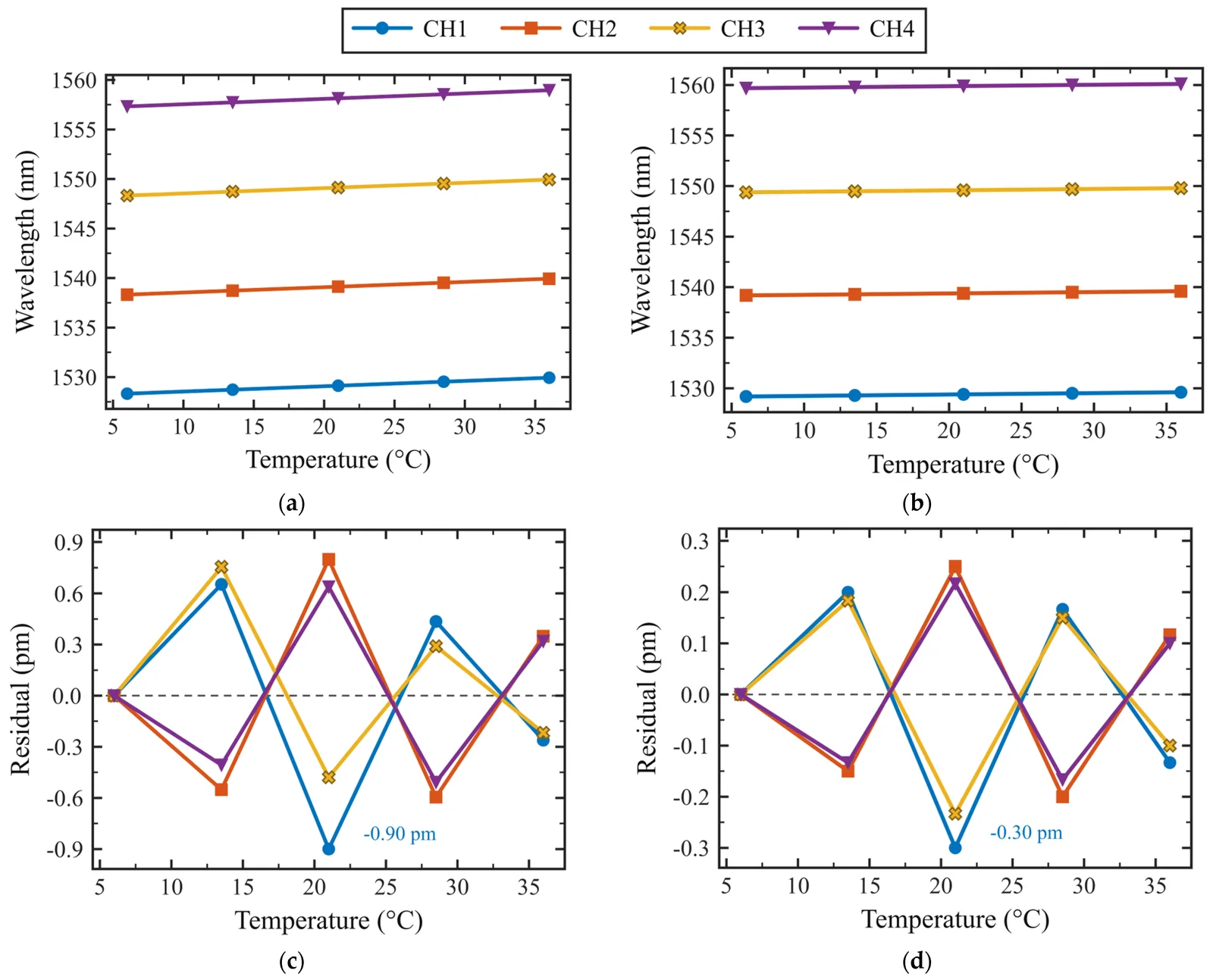
Measured Bragg-wavelength response over the 6–36 °C calibration interval. (**a**) S1 channels; (**b**) T1 channels; (**c**) S1 residuals to reference lines; (**d**) T1 residuals to reference lines.

Figure 4 plots the measured Bragg-wavelength response λ(T) for the four S1 and four T1 channels over the 6–36 °C calibration interval. The five-point records lie on straight lines with the slopes of Table 2, and the largest residual from the fitted lines is 0.9 pm for S1 and 0.3 pm for T1, i.e., below 0.02% of the full-scale wavelength excursion. The first-order model therefore loses negligible information over this range: for the fused-silica FBG the quadratic thermo-optic term contributes about 2 × 10^−4^ pm/°C^2^ (at most 0.12 pm over a 24 °C excursion), the hydrostatic quadratic term is below 10^−2^ pm/MPa^2^, and the mixed ΔT·ΔP term is below 10^−3^ pm/(°C·MPa), so discarding the ΔT^2^, ΔP^2^ and ΔT·ΔP terms changes the strain estimate by less than 0.1 με over the calibrated envelope. Higher-order terms would become significant only for ranges far exceeding those used here.

The eleven applied-strain/readout pairs over 0–10,000 με gave a recalculated root-mean-square error of 9.65 με and a maximum absolute error of 20.67 με. The root-mean-square error was calculated as the square root of the mean squared difference between the readout and the applied strain. A 10 min wavelength standard deviation of 0.8523 pm corresponds to 0.687 με, or 0.0069% of the 10,000 με range. These bench measurements characterize sensor readout performance; the embedded-host strain uncertainty also includes the transfer and compensation terms developed below.

The displacement-stage calibration described above characterizes the bare-fiber sensitivity *K*_ε_, whereas a separate embedded-compression calibration, in which a known axial strain was applied to the cured specimen while the gratings were interrogated, gave a mean grating strain transfer efficiency $\bar{\eta }$ = *a_S_*/*K*_ε_ = 0.80 ± 0.02 (*a_S_* = 0.9924 pm/με). The residual mechanical response of the capillary-isolated temperature channel was *a_T_* = 0.0258 pm/με, i.e., *a_T_*/*a_S_* = 2.6%, which combines with the measured slope ratio *c* ≈ 3.9 to give a weighted leakage χ = 0.10. The residual pressure coefficients were *q_S_* = −3.1 pm/MPa for the bonded strain channel and *q_T_* = −0.4 pm/MPa for the capillary-isolated temperature channel. The T1 temperature was computed from the reference wavelength shift as T = *T*_0_ + Δ*λ_T_*/*b_T_* using *b_T_* ≈ 13.9 pm/°C; the *a_T_* and *q_T_* terms instead contribute approximately 8.2 °C and 1.3 °C, respectively, to the absolute reading at depth ($\left|a_{T}\varepsilon \right|/b_{T}$ = 0.0258 pm/με × 4.4 × 10^3^ με / 13.9 pm/°C ≈ 8.2 °C, and $\left|q_{T}p\right|/b_{T}$ = 0.4 pm/MPa × 46 MPa / 13.9 pm/°C ≈ 1.3 °C), so the T1 value is an apparent temperature rather than a clean internal temperature. These contributions are negligible for the stage increments used in Section 3, because Δp≈0 and the mechanical strain increment is only a few tens of microstrain.

The embedded strain transfer and residual pressure coefficients were obtained from two separate calibrations using the same FBG interrogator as the sea trial (Luna HYPERION si155 (Luna Innovations, Roanoke, VA, USA), 1460–1620 nm, 1 pm accuracy, 10 Hz full-spectrum scan rate). The embedded strain transfer efficiency ($\bar{\eta }$ = 0.80 ± 0.02) and the residual mechanical response of the capillary-isolated temperature channel ($a_{T}$ = 0.0258 pm/με, i.e., 2.6% leakage) were obtained from an embedded-compression calibration, in which a known axial compressive strain was applied stepwise to the cured, instrumented specimen while all gratings were interrogated. Dividing the embedded strain-channel slope $\Delta \lambda_{S}/\Delta \varepsilon$ by the bare-fiber sensitivity $K_{\varepsilon }$ = 1.2405 pm/με (from the eleven-point, 0–10,000 με displacement-stage calibration) gave the transfer efficiency, and the capillary-isolated channel’s wavelength shift per unit applied strain $\Delta \lambda_{T}/\Delta \varepsilon$ gave the residual mechanical response. The residual pressure coefficients (*q_S_* = −3.1 pm/MPa for the bonded strain channel and *q_T_* = −0.4 pm/MPa for the capillary-isolated temperature channel) were obtained from a hydrostatic pressure calibration, in which the specimen was pressurized stepwise in a hydrostatic pressure vessel at constant temperature (referenced to a platinum resistance thermometer, Fluke 5628-12-P (Fluke Corporation, Everett, WA, USA), calibration uncertainty 0.006 °C at 0 °C), and the wavelength shift per unit pressure Δλ/Δp was recorded for both channel types.

### 2.4. Optical Observation Model and Two-Channel Decoupling

The Bragg wavelength depends on the effective refractive index $n_{\mathrm{eff}}$ and grating period Λ [9]:

(1) $$\lambda_{B}=2n_{eff}\cdot \Lambda$$

For small perturbations about a reference state, the embedded-channel response is approximated by

(2) $$\Delta \lambda_{i}=a_{i}\varepsilon_{m}^{mech}+b_{i}\Delta T_{i}+q_{i}\Delta p,\;a_{i}=K_{\varepsilon ,i}{\bar{\eta }}_{i}$$

Here, $\varepsilon_{m}^{\mathrm{mech}}$ is the axial mechanical strain of the homogenized foam host. The effective embedded sensitivity is $a_{i}=K_{\varepsilon ,i}{\bar{\eta }}_{i}$, where $K_{\varepsilon ,i}$ is the fiber strain sensitivity and ${\bar{\eta }}_{i}$ is the mean grating transfer efficiency. The coefficient $b_{i}$ includes optical temperature sensitivity and thermally induced strain transmitted under the calibration constraints. The coefficient $q_{i}$ describes optical and packaging pressure response excluded from the host strain term. Wavelength shifts are in pm and mechanical strain in με; pressure units must match those used for $q_{i}$. For clarity, in Equation (2) ΔT and Δp denote the temperature change and the hydrostatic pressure change in a channel relative to the reference state; the subscript i on the coefficients denotes the channel index.

Both $q_{i}$ and $b_{i}$ are properties of the embedded assembly over the calibrated operating range. Direct pressure response [15] and thermally induced strain transfer [33] make them dependent on packaging and mechanical constraints. Equation (2) therefore uses constant coefficients as a local linear model.

For S and T channels sharing a temperature increment and host mechanical strain, the pressure-corrected observations satisfy [10,11]:

(3) $$\left[\begin{array}{l} y_{S}\\ y_{T} \end{array}\right]=\left[\begin{array}{cc} a_{S} & b_{S}\\ a_{T} & b_{T} \end{array}\right]\left[\begin{array}{l} \varepsilon_{m}^{mech}\\ \Delta T \end{array}\right],\;y_{i}=\Delta \lambda_{i}-q_{i}\Delta p$$

The inverse retains the residual mechanical sensitivity of the temperature channel:

(4) $$\varepsilon_{m}^{mech}=\frac{b_{T}y_{S}-b_{S}y_{T}}{D},\;\Delta T=\frac{a_{S}y_{T}-a_{T}y_{S}}{D},\;D=a_{S}b_{T}-a_{T}b_{S}$$

The determinant *D* controls algebraic invertibility. For $a_{T}=0$ and $b_{T}\neq 0$, the temperature estimate reduces to $y_{T}/b_{T}$; otherwise, both observations enter the inverse. Accurate use also requires common temperature, common host strain, and stable calibration coefficients. These conditions connect the matrix model to the calibration challenges described for optical and composite sensing [34,35,36].

### 2.5. Finite-Grating Strain Transfer

A concentric fiber–coating shear-lag model describes axial transfer under approximately uniform host strain [12,13]. The effective bonded length is 2*L*, $r_{f}$ and $r_{c}$ are the fiber and coating radii. The fiber modulus is $E_{f}$, and the coating shear modulus is $G_{c}$. Linear elasticity, continuous interfaces, and negligible perturbation of the far-field host give

(5) $$\frac{d^{2}\varepsilon_{f}}{dx^{2}}-\beta^{2}\varepsilon_{f}=-\beta^{2}\varepsilon_{m},\;\beta^{2}=\frac{2G_{c}}{E_{f}r_{f}^{2}\ln\left(r_{c}/r_{f}\right)}$$

With zero axial force at the ends of the idealized bonded segment, the local transfer efficiency is

(6) $$\eta \left(x\right)=\frac{\varepsilon_{f}\left(x\right)}{\varepsilon_{m}}=1-\frac{\cosh\left(\beta x\right)}{\cosh\left(\beta L\right)}$$

Averaging over a centered grating of length $l_{g}$ gives

(7) $${\bar{\eta }}_{g}=1-\frac{2\sinh\left(\beta l_{g}/2\right)}{\beta l_{g}\cosh\left(\beta L\right)},\;{\bar{\eta }}_{2L}=1-\frac{\tanh\left(\beta L\right)}{\beta L}$$

For $0<l_{g}\leq 2L$, Equation (7) approximates the tracked wavelength shift using a uniformly weighted mean fiber strain. Its boundary condition is zero axial force at the modeled bond ends. Extended bonding or nonuniform host strain requires the corresponding transfer solution [14,37]. The dimensionless analysis varies βL and $l_{g}/\left(2L\right)$ to isolate their geometric effects; it does not estimate these quantities for the sea-trial specimen.

### 2.6. Hydrostatic Time Dependence and Internal Thermal Lag

For an initially unloaded, isotropic linear viscoelastic host, the bulk creep compliance $J_{K}$ relates hydrostatic pressure to an axial strain component. Taking pressure as positive and compression as negative gives

(8) $$\varepsilon_{m}^{mech}\left(t\right)=-\frac{1}{3}\int_{0}^{t}J_{K}\left(t-\xi \right)dp\left(\xi \right),\;\;J_{K}\left(t\right)=\frac{1}{K_{0}}+\sum_{k=1}^{N}J_{K}\left[1-\exp\left(-t/\tau_{k}\right)\right]$$

Here, $K_{0}$ is the instantaneous bulk modulus, while $J_{k}\geq 0$ and $\tau_{k}>0$ are compliance increments and retardation times. The elastic limit is $\varepsilon_{m}^{\mathrm{mech}}=-p/\left(3K_{0}\right)$. Strain in Equation (8) is dimensionless and is multiplied by $10^{6}$ when expressed in με. This reference model assumes isothermal linear viscoelasticity with fixed material parameters.

Internal temperature is governed by transient conduction and a convective surface boundary:

(9) $$\rho_{m}c_{p}\frac{\partial T}{\partial t}=\nabla \cdot \left(k\nabla T\right),\;-k\frac{\partial T}{\partial n}=h\left(T_{S}-T_{\infty }\right),\;\alpha_{d}=\frac{k}{\rho_{m}c_{p}}$$

The quantities $\rho_{m}$, $c_{p}$, *k*, and *h* denote density, specific heat capacity, thermal conductivity, and surface heat-transfer coefficient, respectively. The temperatures $T_{s}$ and $T_{\infty }$ refer to the surface and ambient water, and $\alpha_{d}$ is thermal diffusivity. During station keeping at approximately constant ambient temperature, a dominant thermal mode gives

(10) $$\theta \left(t\right)=\frac{T\left(t\right)-T_{\infty }}{T\left(t_{0}\right)-T_{\infty }}\approx \exp\left[-\left(t-t_{0}\right)/\tau_{T}\right],\;t_{90}=\tau_{T}\ln10$$

The thermal time constant depends on sensor location, specimen geometry, and the surface boundary condition. A dominant mode reaches 90% of its total temperature change after $2.303\tau_{T}$. The optical conversion must use calibration over the relevant temperature interval [38]. Equations (8) and (10) motivate the competing temporal basis functions examined in Section 4.3. Fitting the observed decay of the internal–seawater temperature difference during station keeping (11.5 °C to 7.7 °C over 4 h) to Equation (10) gives a dominant thermal time constant $\tau_{T}$ ≈ 9.8 h and a local cooling rate of about 0.8 °C/h near the end of the hold. This slow approach to equilibrium confirms that the proposed temperature mismatch limits are achievable only with closely co-located and synchronously sampled paired gratings; a conservative timing analysis in Section 4.5 uses a higher assumed rate of 0.01 °C/s.

## 3. Results

### 3.1. Channel Consistency

The mean temperature slopes were 53.910 pm/°C for S1 and 53.918 pm/°C for S2. Their sample standard deviations were 0.393 and 0.121 pm/°C, respectively, corresponding to coefficients of variation of 0.729% and 0.224%. The mean slopes differed by approximately 0.014%, although S1 showed greater channel dispersion. The eight reference temperature-error gains ranged from 43.23 to 43.93 με/°C. This consistency concerns thermal response; embedded mechanical sensitivities also depend on strain transfer.

### 3.2. Descent and Station Keeping

**Figure 5:**
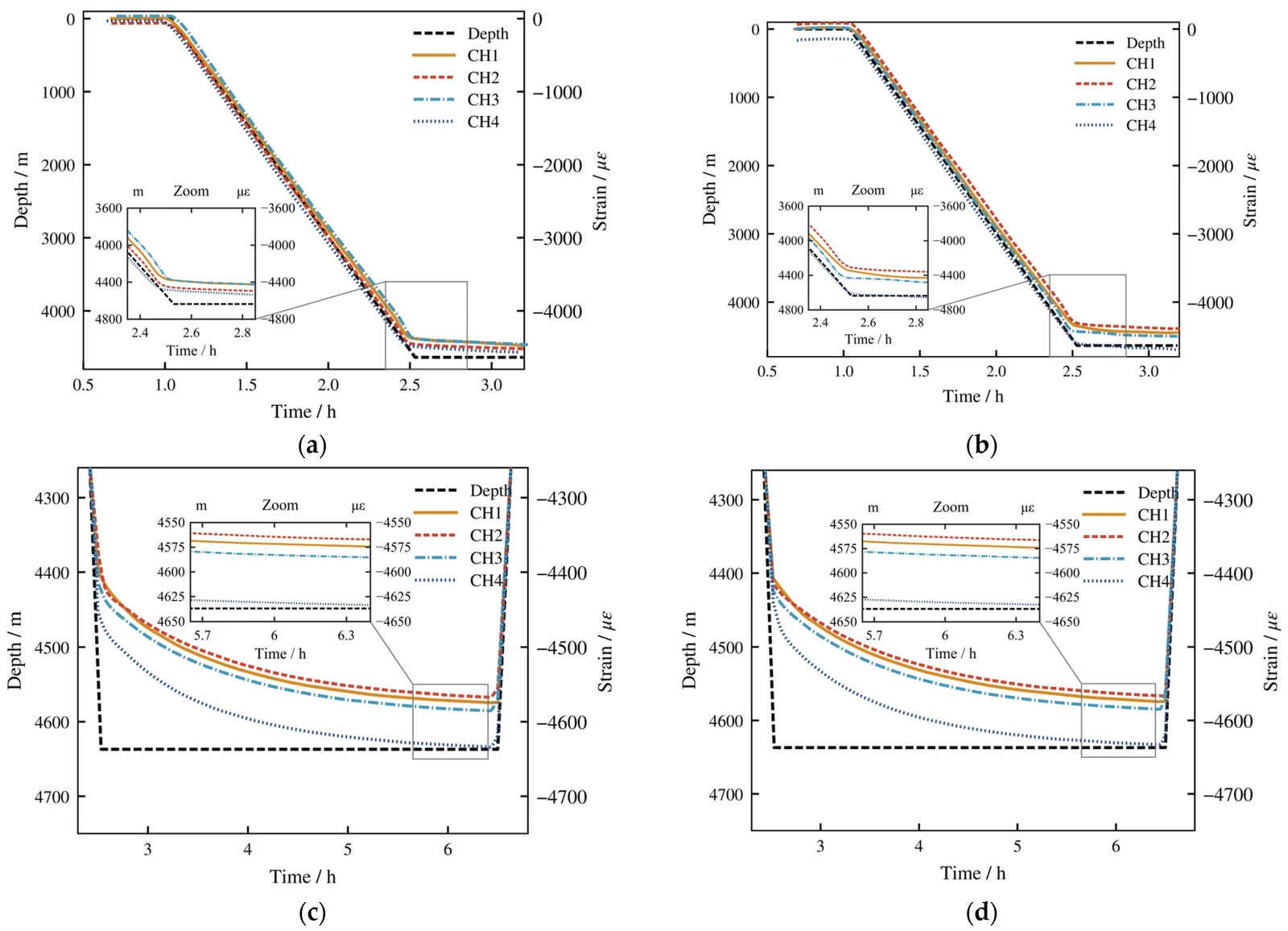
Depth and optical strain indication during descent and station keeping. (**a**,**b**) S1 and S2 during descent; (**c**,**d**) S1 and S2 during station keeping. S2 is shown as an optical indication and is excluded from quantitative paired-channel correction.

During descent, the optical strain indications became increasingly compressive and reached approximately −4.4 × 10^3^ με (Figure 5a,b). S1 and S2 had comparable indication magnitudes. During station keeping, compression continued to increase at a decreasing rate, with channel increments of order 10^2^ με (Figure 5c,d). S2 is used descriptively because its T2 reference was compromised; the stage correction below uses S1/T1. Panels (c) and (d) are expanded views of the station keeping interval (2.5–6.5 h); the continued increase in compression above 6 h reflects viscoelastic creep and internal cooling at nearly constant depth.

Taking the start of station keeping as a local reference separates the stage increment from the accumulated descent response. For synchronized wavelength, temperature, and pressure observations, the channel residual is

(11) $$R_{i}\left(t\right)=\Delta \lambda_{i}\left(t\right)-\Delta \lambda_{i}\left(t_{0}\right)-b_{i}\left[T_{i}\left(t\right)-T_{i}\left(t_{0}\right)\right]-q_{i}\left[p\left(t\right)-p\left(t_{0}\right)\right]$$

For stable coefficients and correctly removed thermal and pressure terms, $R_{i}/a_{i}$ equals the host mechanical strain increment. Table 3 instead applies the nominal paired correction *r* defined in Section 2.2. Under common host strain, substitution of Equation (2) gives $K_{\varepsilon }r=\left(a_{S}-a_{T}\right)\delta \varepsilon_{m}^{\mathrm{mech}}+\left(q_{S}-q_{T}\right)\delta p+b_{S}\delta T_{\mathrm{pair}}$. Here, $\delta T_{\mathrm{pair}}=\delta T_{S}-\delta T_{T}$ measures the difference between the two stage temperature changes. This relation identifies the mechanical, residual pressure, and temperature mismatch terms retained in *r*. For the valid S1/T1 pairs, nominal thermal correction accounted for 76.7% of the channel-summed station keeping indication increment. The remaining increments ranged from −20.2 to −50.7 με. Because the four channels share a single host and deployment history, their fractions are correlated, and the summed ratios are reported as descriptive point estimates rather than as statistically independent replicates.

### 3.3. Ascent and Deck Recovery

**Figure 6:**
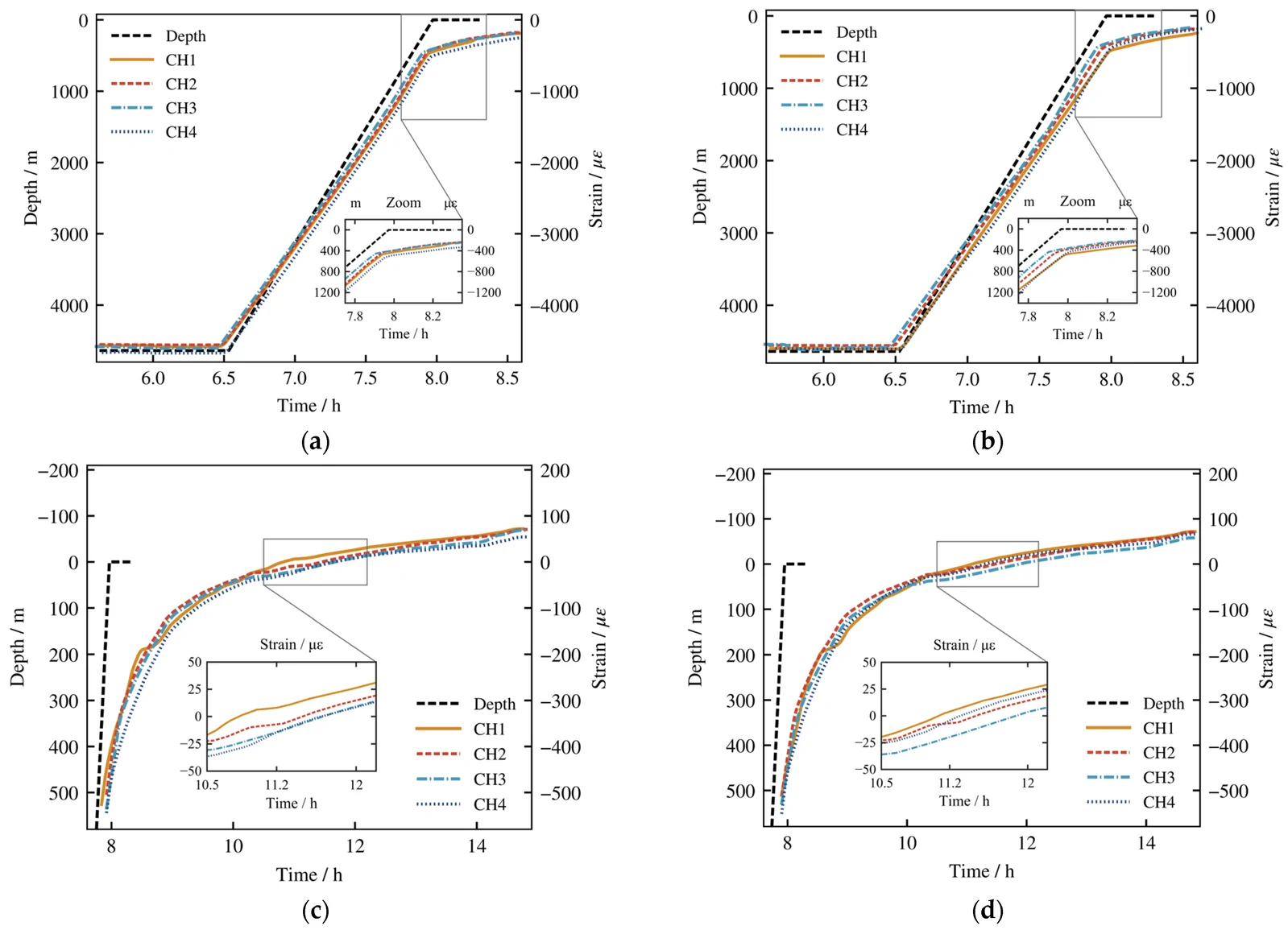
Depth and optical strain indication during ascent and deck recovery. (**a,b**) S1 and S2 during ascent; (**c**,**d**) S1 and S2 during deck recovery. Each panel retains four optical channels and the depth trace on the acquisition time coordinate.

During ascent, the optical strain indication moved toward zero as pressure decreased, retaining a compressive offset at the end of unloading (Figure 6a,b). Deck recovery was initially rapid and then slowed. The curves crossed zero at approximately 11–12 h on the acquisition time coordinate and subsequently became positive (Figure 6c,d).

For S1/T1, nominal thermal correction accounted for 88.0% of the channel-summed four-hour deck recovery increment, leaving +52.9 to +63.8 με across the channels. The zero crossing therefore occurred while thermal and mechanical contributions were still evolving. Mechanical recovery is evaluated at matched pressure and temperature, whereas buoyancy recovery additionally requires displaced-volume and mass measurements. Pressure-induced water uptake provides a separate route to buoyancy change [4]. Table 3 resolves the indication, nominal thermal term, and residual for both stages. Station keeping spans 2.5–6.5 h and deck recovery 7.9–11.9 h on the acquisition coordinate. Each increment is end minus start; for deck CH2, 26.7 − 14.2 = 12.5 °C. The nominal thermal term is *g*δ*T_stage_*, and the residual is *r* = δ$\varepsilon_{eq}$ − *g*δ*T_stage_*. Calculations use the unrounded *g* values from Table 2. The ratios of summed thermal terms to summed indication increments are 76.70% and 87.99%. The per-channel thermal fraction is *f* = *g*δ*T_stage_*/δε_eq_, with *g*, δ*T_stage_* and δε_eq_ taken from Table 2 and Table 3; its standard uncertainty *u*(*f*) is propagated from three inputs: the equal-subtraction gain *u*(*g*) = 0.05 με/°C, the stage temperature change *u*(δ*T_stage_*) = 0.10 °C, and the optical strain indication increment *u*(δε_eq_) = 1.0 με. The gain uncertainty combines the standard uncertainties of the S- and T-channel thermal-slope calibrations and of *K_ε_* = 1.2405 pm/με; the temperature uncertainty combines the T1 calibration residual with the acquisition noise over each stage; and the strain uncertainty is the 10 min wavelength standard deviation 0.8523 pm (0.687 με) rounded to 1.0 με for the endpoint difference. First-order propagation yields the 0.7–2.2 percentage-point uncertainties listed in the last column.

### 3.4. Thermal Contributions to Stage Response

**Figure 7:**
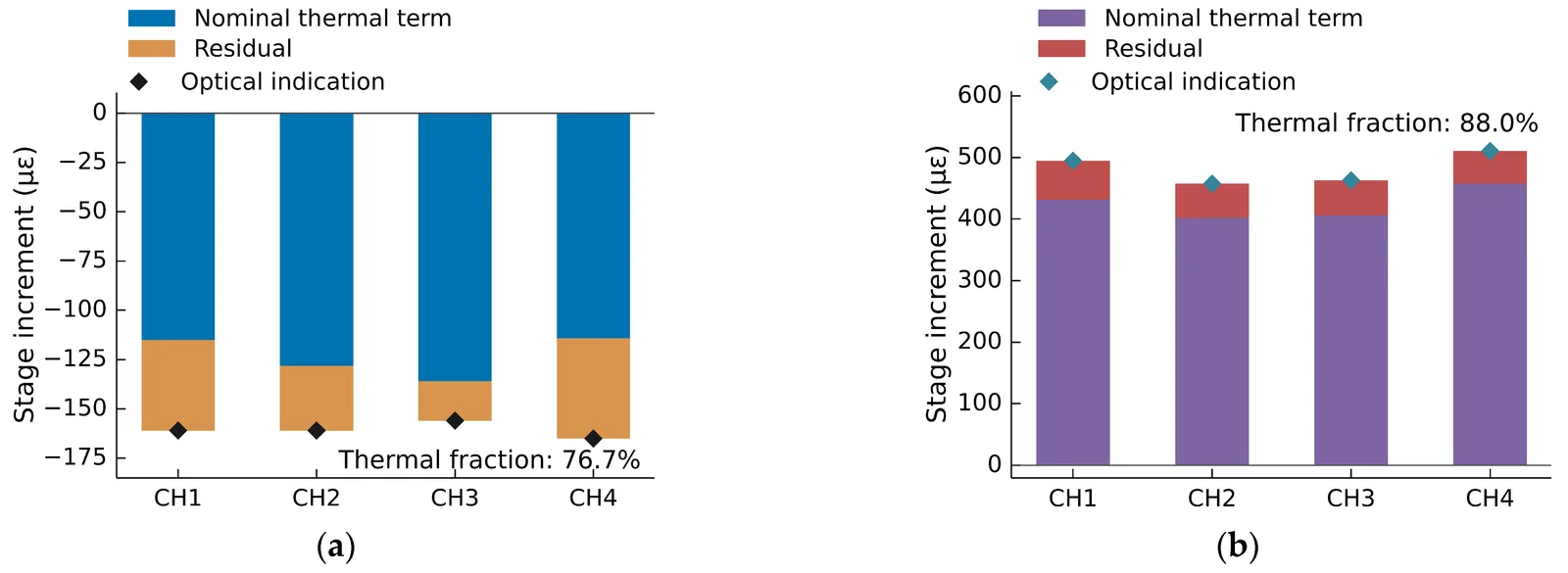
Decomposition of reported S1/T1 stage increments using the nominal temperature correction. (**a**) Station keeping; (**b**) deck recovery. Stacked bars show the calculated thermal term and remaining residual; black diamonds mark the reported optical indication increment. Values are calculated from Table 3 with *K*_ε_ = 1.2405 pm/με. The figure displays stage endpoints and does not interpolate a temperature history.

**Figure 8:**
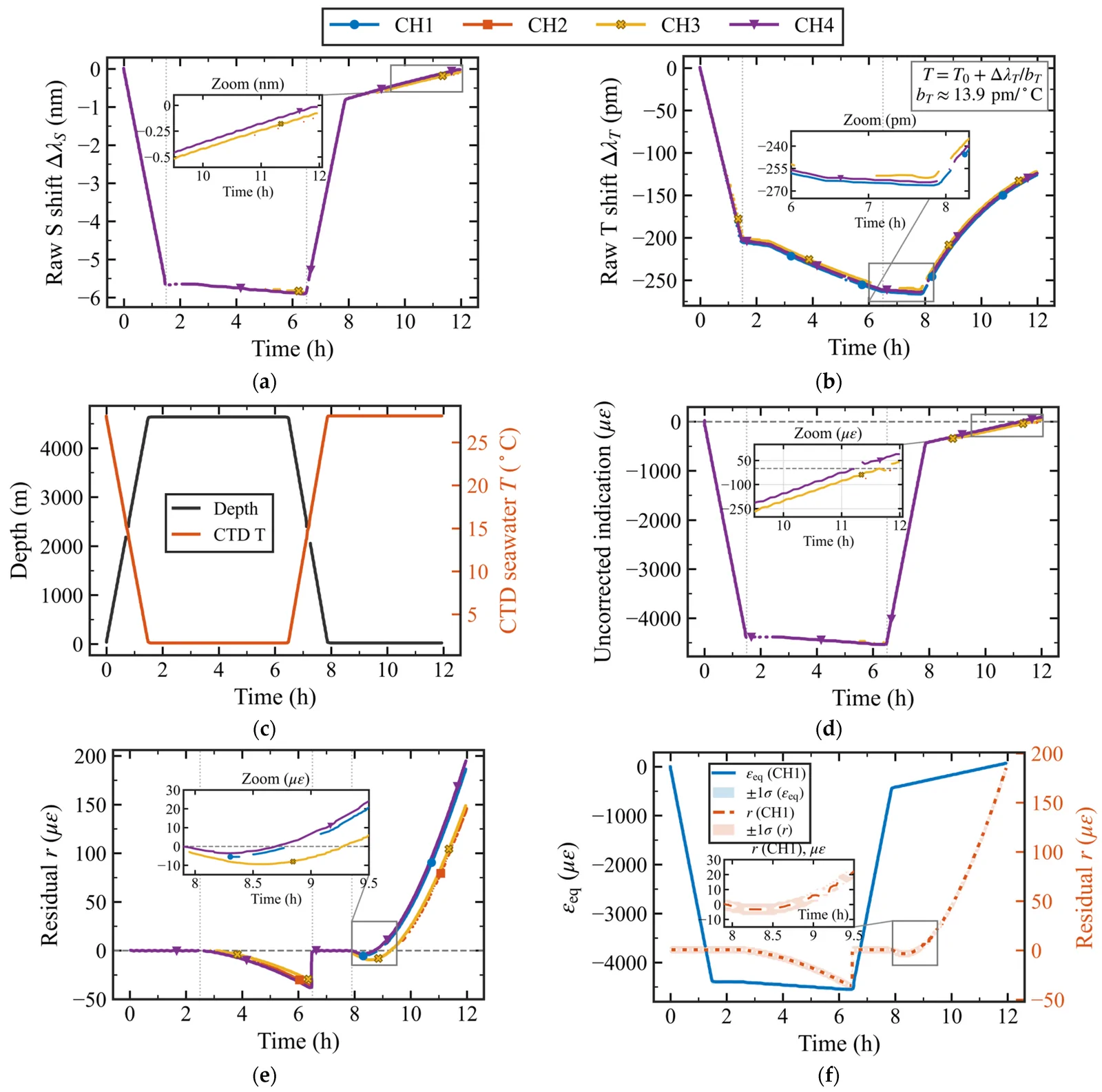
Channel-resolved deployment records for S1/T1 at 4637 m. (**a**) Raw S-channel wavelength shift; (**b**) raw T-channel wavelength shift and the conversion *T* = *T*_0_ + Δ*λ*_T_/*b*_T_; (**c**) CTD temperature and depth; (**d**) uncorrected optical strain indication; (**e**) nominally corrected residual; (**f**) associated ±1σ uncertainty bands.

The mean T1 temperature for the valid station keeping interval decreased from 13.075 to 9.250 °C while the CTD seawater temperature remained approximately 1.6 °C. The mean internal temperature therefore still exceeded the seawater temperature by approximately 7.7 °C after the four-hour hold. This persistent thermal disequilibrium explains why the optical response continued to evolve at nearly constant depth. The mean indication increment was −160.75 με, of which −123.30 με was assigned to the nominal thermal term. During deck recovery, the mean indication increased by 481.00 με, with a nominal thermal contribution of 423.22 με. Figure 7 summarizes these channel-resolved stage contributions from Table 3. Per-channel thermal fractions and their propagated uncertainties are tabulated in Table 3, and Figure 8 shows the channel-resolved time histories of the raw S and T wavelength shifts, the CTD temperature and depth, the uncorrected optical strain, the nominally corrected residual, and the associated uncertainty bands.

Continued internal cooling during station keeping and reheating on deck produced thermal terms with opposite signs. Their magnitudes dominated the respective indication increments, explaining why a slowly changing optical signal cannot be assigned to viscoelastic recovery alone. The smaller residuals make strain transfer stability and local S–T temperature matching consequential, as quantified in the following error analysis.

## 4. Analytical Results and Discussion

### 4.1. Spatial Averaging and Effective Mechanical Sensitivity

**Figure 9:**
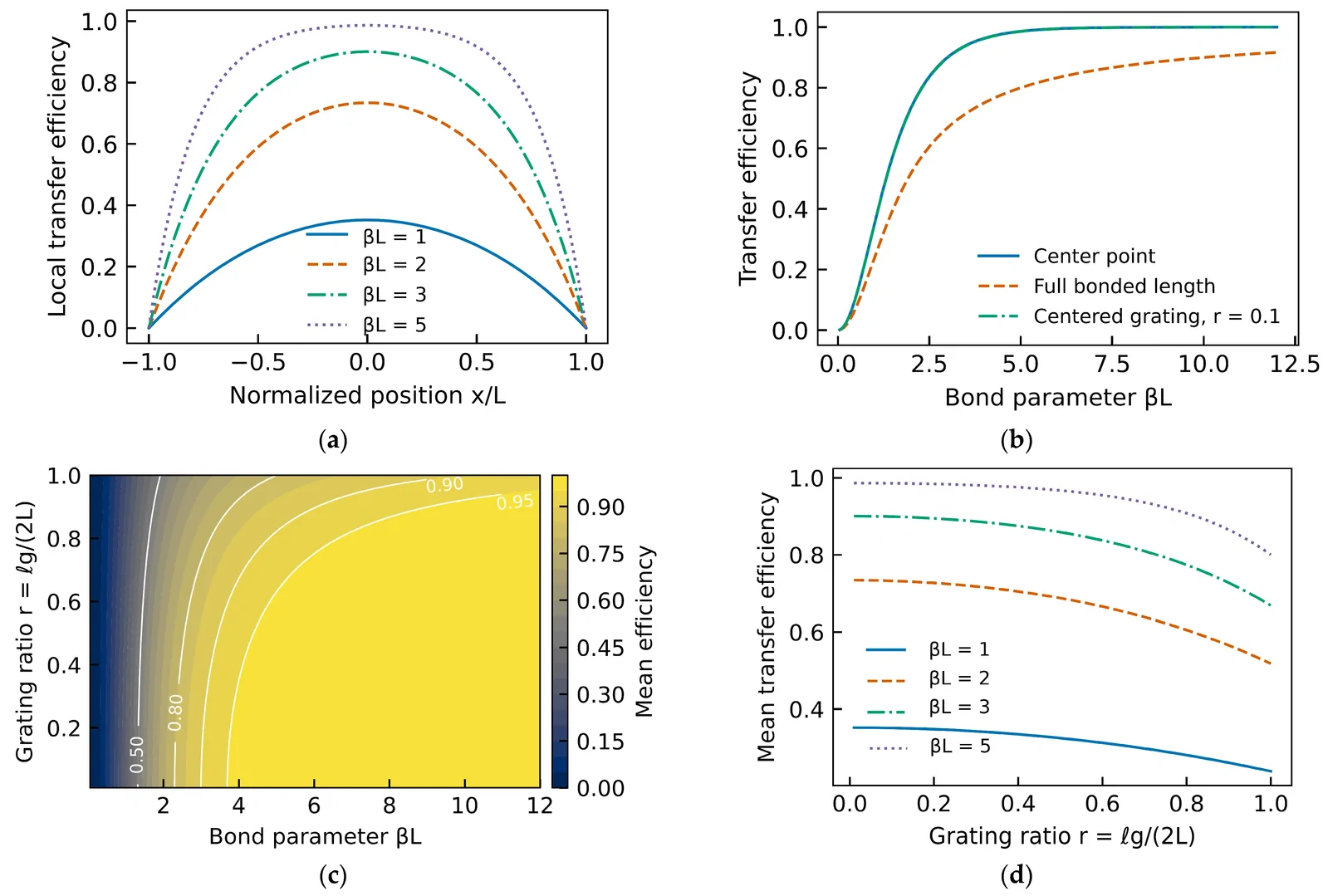
Calculated finite-grating strain transfer with zero axial force at the bond ends. (**a**) Local profiles; (**b**) center-point and grating-averaged efficiencies; (**c**) mean efficiency versus bond parameter and relative grating length; (**d**) grating-length dependence. All panels follow Equations (6) and (7).

Figure 9 evaluates the local and grating-averaged transfer efficiencies in Equations (6) and (7). At βL=3, the center-point efficiency is 0.901, whereas the full-bonded-length mean is 0.668. At βL=5, the corresponding values are 0.987 and 0.800. A grating extending toward the bond ends therefore averages regions with substantial attenuation even when its center closely follows host strain. Increasing βL or shortening the centered grating improves mean transfer through distinct geometric effects.

For $\bar{\eta }<1$, using the bare-fiber sensitivity directly underestimates host strain magnitude and overestimates a modulus inferred from that strain. Grating fabrication controls length and spectrum [39], while the cured host–fiber assembly determines effective mechanical sensitivity. Channel comparison therefore requires calibrated sensitivities as well as wavelength amplitudes.

### 4.2. Temperature Mismatch and Uncertainty Propagation

Let $\delta T_{i}$ be the strain-grating temperature minus the temperature used for compensation. With pressure response and sensitivities fixed, Equation (2) gives the strain bias and its single-contribution bound:

(12) $$\delta \varepsilon_{T,i}=\frac{b_{i}}{a_{i}}\delta T_{i},\;\left|\delta T_{i}\right|\leq \frac{a_{i}}{\left|b_{i}\right|}\varepsilon_{tol}$$

**Figure 10:**
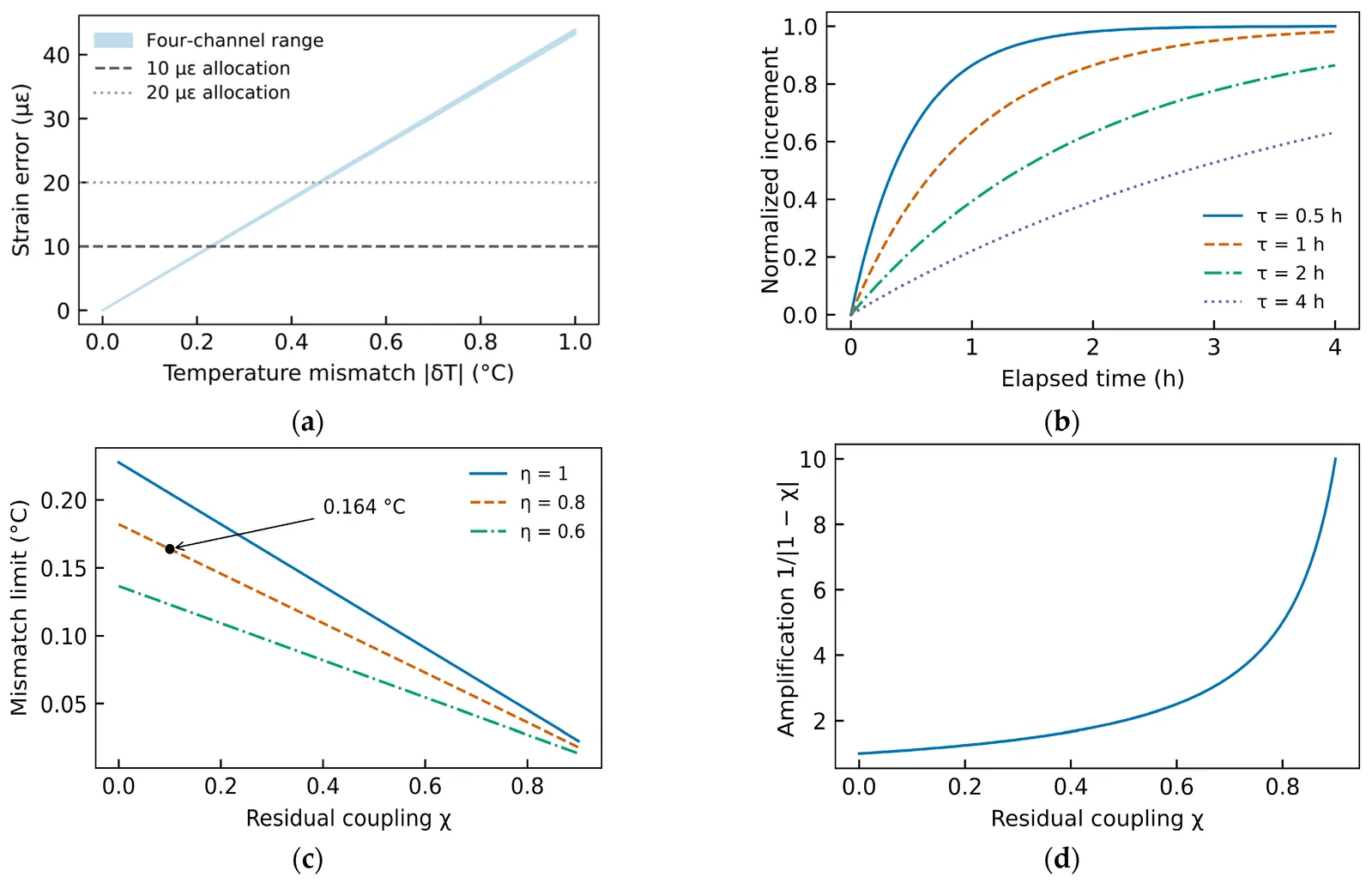
Calculated compensation and transient-response scenarios. (**a**) Temperature-error range for the four S1 slopes at unit transfer; (**b**) normalized exponential increments; (**c**) temperature mismatch limit for a 10 με allocation; (**d**) noise amplification by weighted mechanical leakage. The marked point in (**c**) uses $\bar{\eta }=0.8$ and χ=0.10; its derivation is given in Section 4.5.

At unit transfer, the measured slopes give temperature-error gains of 43.23–43.93 με/°C (Figure 10a). Mismatches of 0.1, 0.5, and 1.0 °C produce approximately 4.3, 21.7, and 43.5 με of error. Using the largest slope, 54.47 pm/°C, a 10 με design allocation permits 0.228 °C mismatch. Doubling the allocation to 20 με permits 0.455 °C. The allocation specifies an allowable bias contribution rather than a measured field accuracy.

For a single strain channel with independently estimated temperature and pressure, first-order propagation of independent input uncertainties gives

(13) $$u^{2}\left(\hat{\varepsilon }\right)\approx \frac{u^{2}\left(\Delta \lambda \right)+b^{2}u^{2}\left(\Delta T\right)+q^{2}u^{2}\left(\Delta p\right)+{\left(\Delta T\right)}^{2}u^{2}\left(b\right)+{\left(\Delta p\right)}^{2}u^{2}\left(q\right)+{\hat{\varepsilon }}^{2}u^{2}\left(a\right)}{a^{2}}$$

Here, *u* denotes standard uncertainty; the temperature and pressure terms refer to increments from the reference state. Coefficient uncertainty becomes more influential as the temperature or pressure excursion increases. For correlated inputs, the covariance form $u^{2}\left(\hat{\varepsilon }\right)=J\Sigma J^{T}$ retains their joint contribution [11]. Section 4.9 gives the Jacobian for the paired inverse. The between-channel dispersion in Table 2 does not specify this calibration covariance.

### 4.3. Thermal and Viscoelastic Identifiability

Temporal separation requires independent response shapes as well as an invertible optical sensitivity matrix [18,19,20]. To examine this requirement, consider fixed thermal and viscoelastic time constants during a constant-pressure interval. Before thermal correction, the optical indication increment $\delta \varepsilon_{\mathrm{eq}}\left(t\right)$ is approximated by

(14) $$\delta \varepsilon_{eq}\left(t\right)=A_{T}\left[1-\exp\left(-s/\tau_{T}\right)\right]+A_{v}\left[1-\exp\left(-s/\tau_{v}\right)\right],\;s=t-t_{0}$$

The amplitudes are $A_{T}$ and $A_{v}$, and the time constants are $\tau_{T}$ and $\tau_{v}$. At the sampled times, define $f_{T}=1-\exp\left(-s/\tau_{T}\right)$ and $f_{v}=1-\exp\left(-s/\tau_{v}\right)$. The normalized design matrix is $F=\left[f_{T}/∥f_{T}∥,f_{v}/∥f_{v}∥\right]$, with column similarity and condition number

(15) $$\gamma =\frac{f_{T}^{T}f_{v}}{\left\|f_{T}\right\|\left\|f_{v}\right\|},\;\kappa_{2}\left(F\right)=\sqrt{\frac{1+\left|\gamma \right|}{1-\left|\gamma \right|}}$$

**Figure 11:**
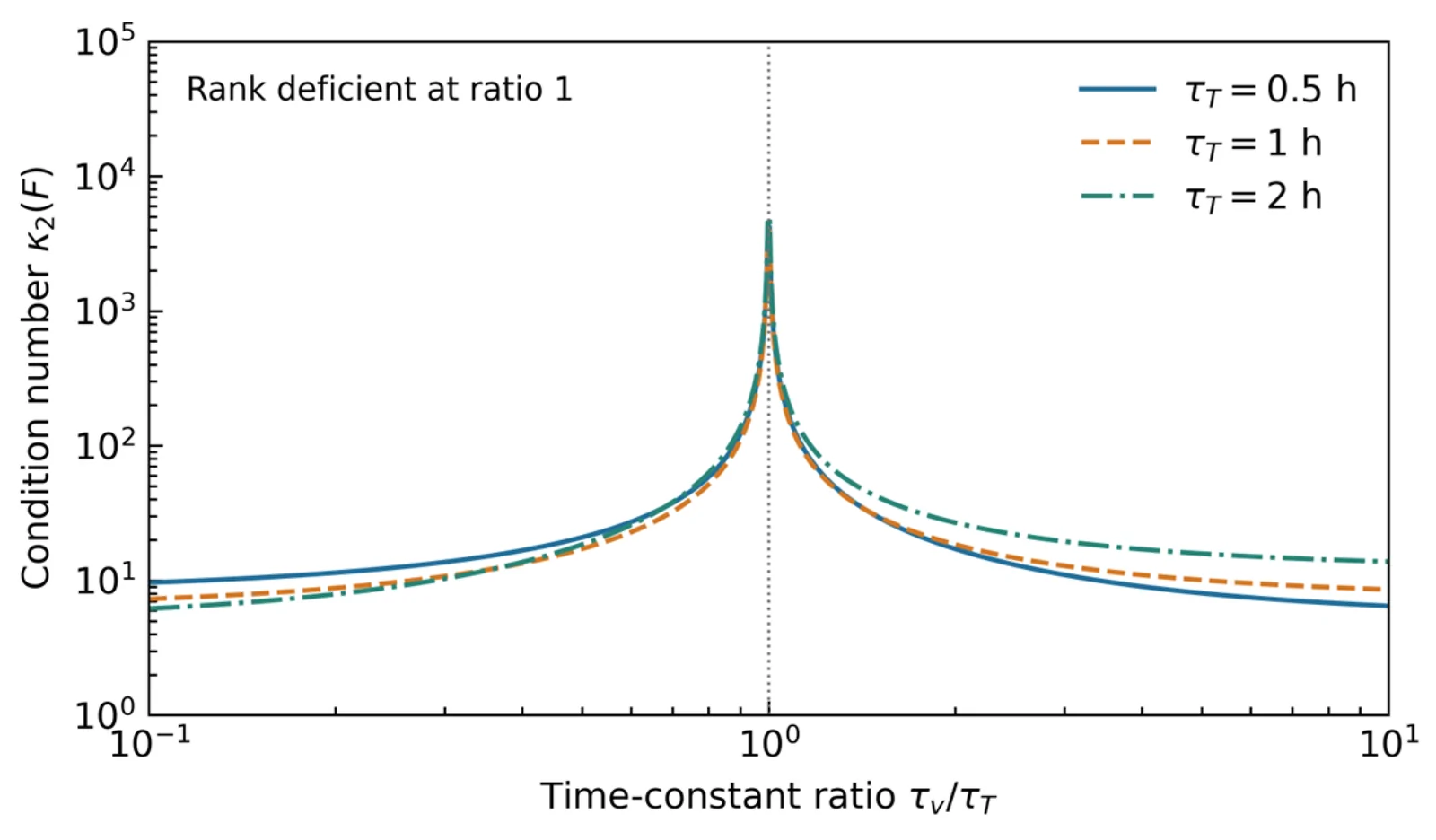
Conditioning of normalized thermal and viscoelastic basis functions. The condition number is calculated at 401 equally spaced points over 4 h for the indicated thermal time constants. The vertical dotted line marks equal time constants and rank deficiency at $\tau_{v}/\tau_{T}=1$

Equal time constants produce identical columns, preventing unique separation of the two amplitudes. Over a 4 h window with $\tau_{T}=2$ h, values of $\tau_{v}=1$, 3, and 4 h give condition numbers of 18.47, 41.64, and 26.82 (Figure 11). These values use 401 equally spaced samples, fixed time constants, and a known zero-increment baseline. Estimating the baseline or time constants introduces additional parameter dependencies. For correlated residuals, the design matrix must be weighted by the noise covariance before assessing statistical precision. For readability, the channel-resolved thermal fractions and their uncertainties are now collected in Table 3 rather than enumerated in the text.

Independent temperature measurements constrain the thermal component, while unloading and isothermal recovery change the mechanical loading history. Longer station keeping improves coverage of slow processes, but identical temporal bases remain inseparable from that record alone. When time constants are estimated, profile likelihood can assess the resulting parameter intervals [18].

A separate coupling arises from residual mechanical response in the temperature channel. Under common temperature and corrected pressure response, neglecting $a_{T}$ gives

(16) $${\hat{\varepsilon }}_{a_{T}=0}=\left(1-\chi \right)\varepsilon_{m}^{mech},\;\chi =\frac{b_{S}a_{T}}{b_{T}a_{S}},\;D=a_{S}b_{T}\left(1-\chi \right)$$

The weighted leakage χ combines mechanical sensitivity with the temperature-sensitivity ratio. Values of 0.05, 0.10, and 0.20 produce corresponding underestimates of mechanical strain magnitude when $a_{T}$ is omitted. A 5% relative-bias allocation therefore requires $\left|\chi \right|\leq 0.05$, or $\left|a_{T}/a_{S}\right|\leq 0.05\left|b_{T}/b_{S}\right|$. For the measured S1/T1 slope ratios, $\left|a_{T}/a_{S}\right|$ must be approximately 1.28–1.31% or less. Thus, a small mechanical response of the loose-tube channel can become appreciable after weighting by the roughly fourfold temperature-sensitivity ratio.

### 4.4. Transfer Stability and Noise Amplification

The preceding transfer model assumes constant $\bar{\eta }$. After removing thermal and direct pressure effects, the fiber mechanical strain is $\varepsilon_{f}=\bar{\eta }\varepsilon_{m}^{\mathrm{mech}}$. Expanding this product about the start of station keeping and retaining first-order terms gives

(17) $$\delta \varepsilon_{f}\approx {\bar{\eta }}_{0}\delta \varepsilon_{m}^{mech}+\varepsilon_{m,0}^{mech}\delta \bar{\eta }$$

Transfer drift contributes to the signal even at constant host strain. At an assumed separated fiber mechanical strain magnitude of 4400 με, a 1% relative transfer change produces 44 με of apparent change. Increased transfer makes compression more negative, decreased transfer shifts it toward zero. This scale calculation shows why interface stability matters when the residual stage response is only tens of microstrain.

For $c=b_{S}/b_{T}$, the common-temperature inverse is $\hat{\varepsilon }=\left(y_{S}-cy_{T}\right)/\left(a_{S}-ca_{T}\right)$. With calibration coefficients fixed, observation noise contributes

(18) $$u^{2}\left(\hat{\varepsilon }\right)=\frac{u^{2}\left(y_{S}\right)+c^{2}u^{2}\left(y_{T}\right)-2c\mathrm{cov}\left(y_{S},y_{T}\right)}{a_{S}^{2}{\left(1-\chi \right)}^{2}}$$

At fixed observation covariance, weighted leakage amplifies standard uncertainty by $1/\left|1-\chi \right|$. The amplification is 1.11, 2, and 10 at χ=0.10, 0.50, and 0.90, respectively. Full inversion corrects the bias from neglecting $a_{T}$, but the estimate remains noise-sensitive as the weighted mechanical sensitivities approach equality.

### 4.5. Joint Temperature-Matching Criterion

Let $\Delta T_{S}$ and $\Delta T_{T}$ be temperature increments relative to the common reference, and define their mismatch as $\delta T=\Delta T_{S}-\Delta T_{T}$. Substituting these increments into the common-temperature inverse, with matched host strain and corrected pressure terms, gives

(19) $$\delta \varepsilon_{T}=\frac{b_{S}\delta T}{a_{S}\left(1-\chi \right)}$$

Temperature mismatch bias is amplified by the same coupling factor as observation noise. Assigning a tolerance $\varepsilon_{\mathrm{tol}}$ to this contribution gives

(20) $$\left|\delta T\right|\leq \frac{\varepsilon_{tol}\left|a_{S}\left(1-\chi \right)\right|}{\left|b_{S}\right|}$$

**Figure 12:**
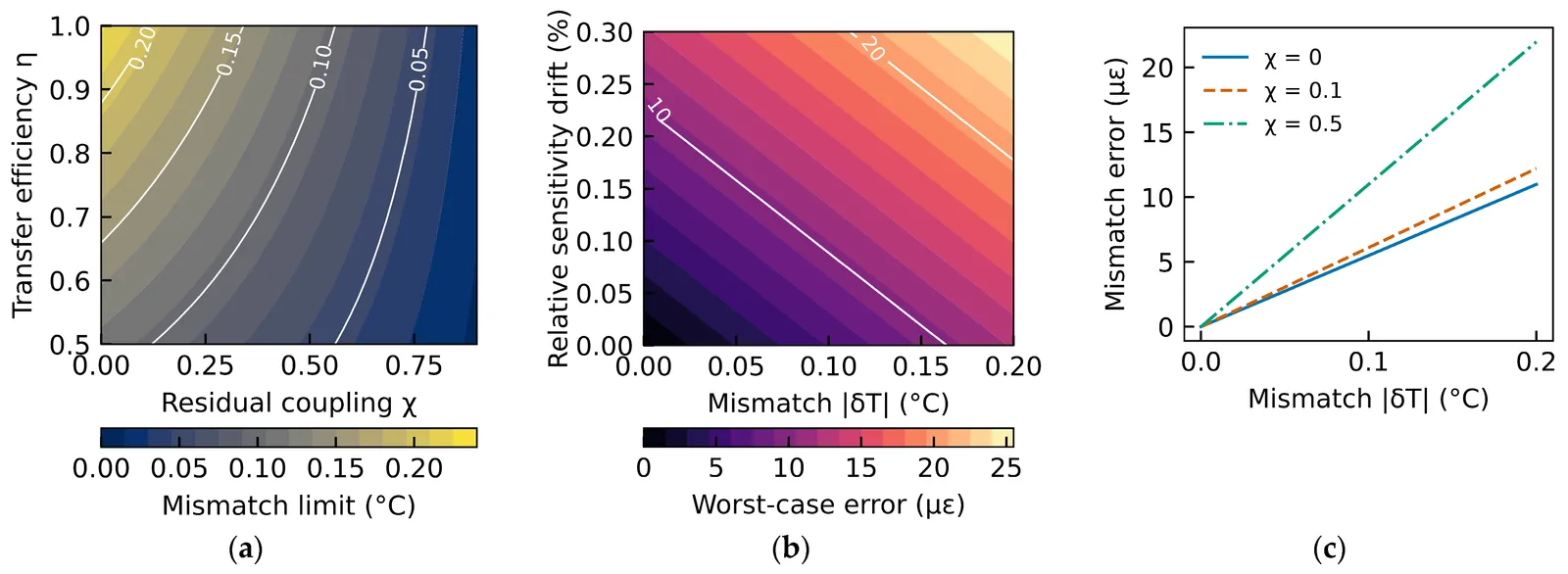
Calculated error allocations. (**a**) Temperature mismatch limit for a 10 με single-contribution budget; (**b**) combined mismatch and sensitivity drift bounds at $\bar{\eta }=0.8$, χ=0.10, and $\left|\varepsilon \right|=4400$ με; (**c**) mismatch-error amplification by weighted leakage at $\bar{\eta }=0.8$.

For $b_{S}=54.47$ pm/°C and $K_{\varepsilon }=1.24$ pm/με, a 10 με allocation permits 0.228 °C mismatch at $\bar{\eta }=1$ and χ=0. At χ=0.10, the limit becomes 0.205 °C. Reducing $\bar{\eta }$ to 0.8 tightens it to 0.164 °C, 28% below the ideal limit. Figure 12a maps this dependence over the analytical parameter range.

Spatial separation and timing offset both contribute to temperature mismatch. Locally, $\delta T\approx \nabla T\cdot \delta x+\dot{T}\delta t$, with consistent signs for the position and timing differences. If the entire 0.164 °C allowance were assigned to an assumed temperature rate of 0.01 °C/s, the timing limit would be 16.4 s. Spatial gradients consume part of that allowance and can also alter thermally induced strain transfer.

### 4.6. Information Available from Hydrostatic Calibration

Hydrostatic loading can also couple parameters that appear separately in the observation model. In the isothermal isotropic elastic limit, substituting host strain into Equation (2) gives

(21) $$\Delta \lambda_{i}=\left(q_{i}-\frac{10^{6}a_{i}}{3K}\right)\Delta p$$

Pressure and bulk modulus *K* use the same units, and $10^{6}$ converts strain to με. An elastic pressure sweep identifies the combined slope $q_{i}-10^{6}a_{i}/\left(3K\right)$. It cannot identify both $q_{i}$ and *K* when both are unknown, regardless of the number of points on that loading path. An uncalibrated $a_{i}$ introduces a further dependency.

Independent bulk-deformation measurements can constrain *K* before residual pressure sensitivity is estimated. Conversely, independent characterization of the optical and packaging pressure response supports subsequent modulus estimation. A mechanically isolated temperature channel constrains its own pressure coefficient, which need not equal that of the bonded strain channel. Each calibration must therefore add an independent physical constraint. For the present specimen, the residual pressure coefficient $q_{T}$ = −0.4 pm/MPa of the capillary-isolated T1 channel contributes ≈1.3 °C to the absolute depth reading at 46 MPa (0.4 pm/MPa × 46 MPa / 13.9 pm/°C), so the field T1 temperatures are reported with this caveat rather than corrected explicitly.

### 4.7. Combined Error Allocation and Observation Design

The mismatch allowance must share an overall budget with effective sensitivity drift, pressure residuals, and measurement noise. Define $A=a_{S}\left(1-\chi \right)$, $c=b_{S}/b_{T}$, and $\delta z=\delta y_{S}-c\delta y_{T}$ for additive observation errors. At fixed *c* and to first order, the absolute strain error is bounded by

(22) $$\left|\delta \varepsilon \right|\leq \left|b_{S}/A\right|\left|\delta T\right|+\left|\varepsilon \right|\left|\delta A/A\right|+\left|\delta z/A\right|$$

Figure 12b evaluates this bound at $\bar{\eta }=0.8$, χ=0.10, and an assumed host strain magnitude of 4400 με. A 0.1% relative drift in *A* contributes 4.4 με, leaving 5.6 με of the 10 με budget. The corresponding temperature mismatch allowance is 0.092 °C before adding pressure and noise terms. Section 4.9 treats uncertainty in *c* through the individual calibration coefficients.

Equation (22) adds worst-case absolute bounds, whereas Equation (13) propagates independent standard uncertainties in quadrature. For sensitivity drift alone, the 10 με budget at 4400 με requires $\left|\delta A/A\right|\leq 0.227\%$. Contributions from temperature mismatch, pressure, or noise tighten this requirement.

**Figure 13:**
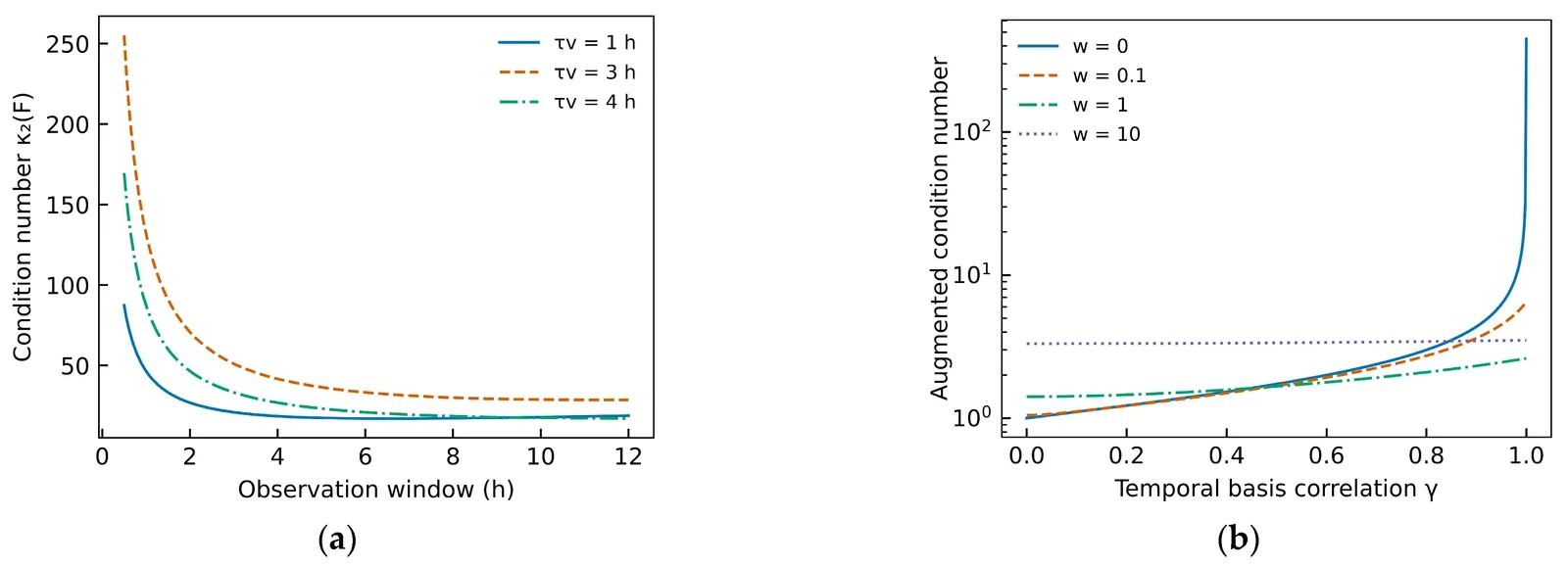
Temporal observation design. (**a**) Condition number versus window duration for *τ_T_* = 2 h and 401 samples per window; (**b**) condition number after adding independent thermal-amplitude information.

Figure 13a varies observation duration while retaining 401 samples per window. Long windows increasingly weight the common plateau, so the normalized-basis condition number can vary nonmonotonically. This comparison changes the sampling schedule; it does not represent adding independent samples to an existing record. Parameter precision also depends on basis-column magnitudes and noise covariance. Independent thermal information directly constrains the competing amplitude.

Adding independent information of relative weight *w* on the normalized thermal amplitude gives the augmented Gram matrix:

(23) $$G=F^{T}F+diag\left(w,0\right)=\left[\begin{array}{cc} 1+w & \gamma \\ \gamma & 1 \end{array}\right],\;\det\left(G\right)=1+w-\gamma^{2}$$

The condition number of a matrix is the ratio of its largest to smallest singular value, $\kappa =\sigma_{\max}/\sigma_{\min}$. For the symmetric augmented Gram matrix the singular values equal the eigenvalues, so the condition number $\kappa =\lambda_{\max}/\lambda_{\min}$ is

(24) $$\kappa =\frac{2+w+\sqrt{w^{2}+4\gamma^{2}}}{2+w-\sqrt{w^{2}+4\gamma^{2}}}$$

For coincident temporal bases, any w>0 restores rank in this two-amplitude model. The weight measures thermal information after consistent normalization and noise weighting. At large *w*, the augmented condition number can increase because the information becomes directionally imbalanced. Section 4.9 shows that mechanical-amplitude variance can nevertheless continue to decrease.

### 4.8. Spatial Strain Mismatch in Paired Channels

Paired gratings can also sample different host mechanical strains. Let $\varepsilon_{S}$ be the target strain and $\varepsilon_{T}$ the host strain driving the temperature channel’s residual mechanical response. With $\delta \varepsilon =\varepsilon_{T}-\varepsilon_{S}$, substitution into the common-strain inverse gives

(25) $$\hat{\varepsilon }-\varepsilon_{S}=\frac{\left(b_{S}/a_{S}\right)\delta T-\chi \delta \varepsilon }{1-\chi },\;\delta \varepsilon =\varepsilon_{T}-\varepsilon_{S}$$

(26) $${\left|\delta T\right|}_{\max}=\frac{\left|a_{S}\right|}{\left|b_{S}\right|}\left[\varepsilon_{tol}\left|1-\chi \right|-\left|\chi \right|{\left|\delta \varepsilon \right|}_{\max}\right]$$

**Figure 14:**
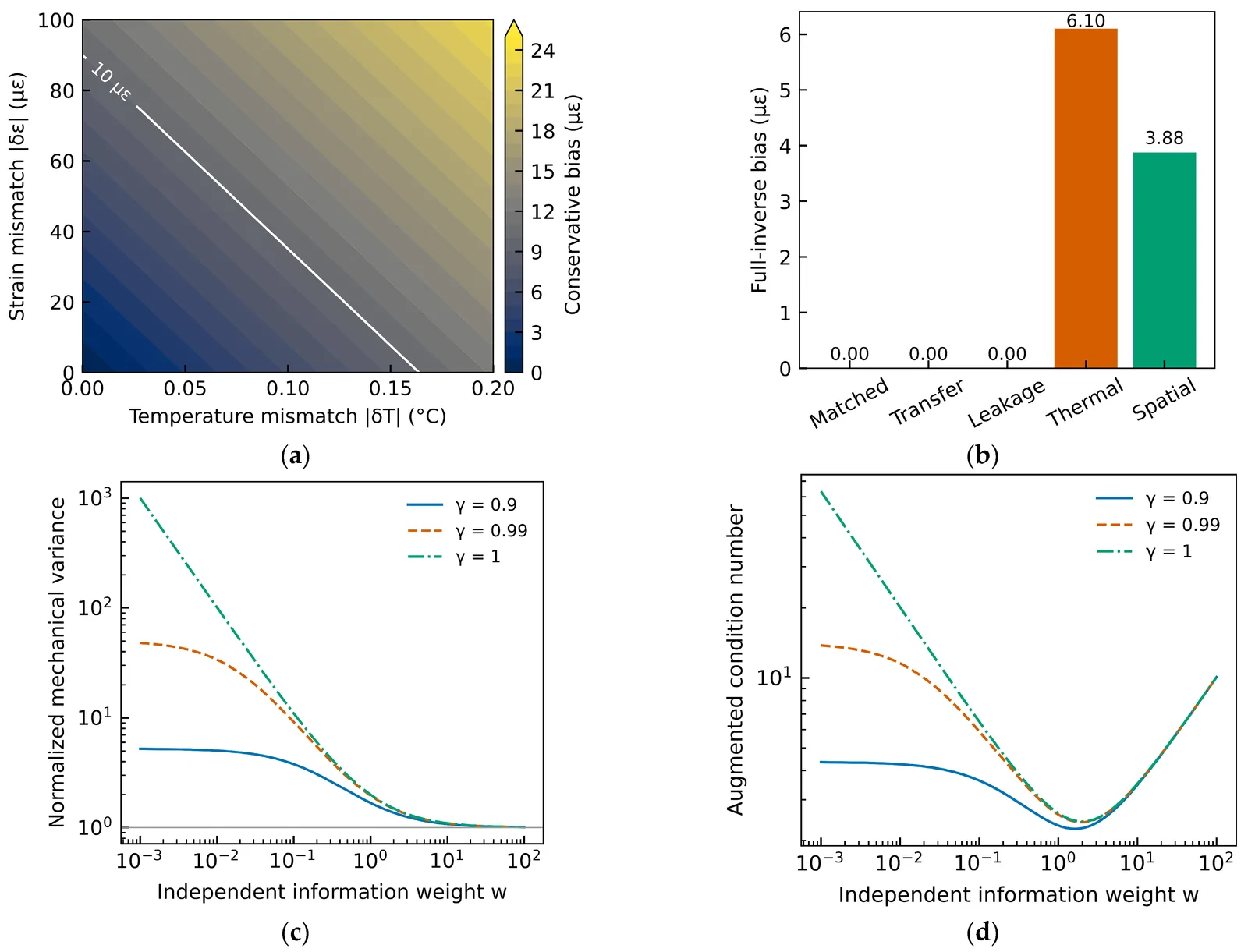
Joint mismatch and information calculations. (**a**) Temperature and strain mismatch budget at $\bar{\eta }=0.8$ and χ=0.10; (**b**) forward–inverse substitution biases, including three matched-input cases; (**c**) mechanical-amplitude variance normalized by *σ*^2^; (**d**) augmented condition number at the same information weights.

Equation (26) allocates bounded temperature and strain mismatch conservatively. Its bracket must be nonnegative; otherwise, strain mismatch alone exhausts the budget. At $\bar{\eta }=0.8$, χ=0.10, and ${\left|\delta \varepsilon \right|}_{\max}=20$ με, strain mismatch contributes 2.22 με. The remaining 7.78 με permits approximately 0.127 °C temperature mismatch (Figure 14a). These two contributions consume the full allocation before additional drift or noise is included.

A forward–inverse substitution checks the algebra using $\varepsilon_{S}=-4400$ με, $\Delta T_{T}=-20$ °C, and an illustrative $b_{T}=12$ pm/°C. Matched inputs recover the imposed strain with reduced transfer and nonzero leakage. At $\bar{\eta }=0.8$ and χ=0.10, a 0.1 °C temperature mismatch produces 6.10 με bias. Adding a spatial strain mismatch of +20 με changes the signed bias to 3.88 με, as predicted by Equation (25). The absolute-bound criterion retains both contributions instead of relying on their cancelation (Figure 14b).

### 4.9. Calibration Covariance and Parameter Precision

The full calibration covariance is obtained by treating the observations and all four sensitivity coefficients as uncertain. With the input vector x and effective sensitivity *A* defined below, differentiation of the inverse gives

(27) $$x=\left(y_{S},y_{T},a_{S},a_{T},b_{S},b_{T}\right),\;A=a_{S}-ca_{T}$$

(28) $$J=\frac{1}{A}\left(1,-c,-\hat{\varepsilon },c\hat{\varepsilon },-\hat{\Delta T},c\hat{\Delta T}\right),\;c=\frac{b_{S}}{b_{T}}$$

(29) $$\hat{\Delta T}=\frac{y_{T}-a_{T}\hat{\varepsilon }}{b_{T}},\;u^{2}\left(\hat{\varepsilon }\right)=J\sum_{x}J^{T}$$

The covariance matrix $\Sigma_{x}$ includes shared calibration and reference errors. For $y_{i}=\Delta \lambda_{i}-q_{i}\Delta p$, the derivatives with respect to Δp, $q_{S}$, and $q_{T}$ are $\left(-q_{S}+cq_{T}\right)/A$, −Δp/A, and cΔp/A. These derivatives propagate the original wavelength and pressure inputs without counting pressure uncertainty twice. Equations (27)–(29) are linearized about matched temperature and host strain. Spatial mismatch remains a separate bias unless a probabilistic model is specified; near A=0, nonlinear uncertainty propagation is needed.

For temporal estimation, let $\alpha_{T}$ and $\alpha_{v}$ be amplitudes of the unit-norm columns of F. Each equals the physical amplitude multiplied by its original basis-column norm. With independent observation noise of variance $\sigma^{2}$ and independent thermal information $w/\sigma^{2}$, the amplitude covariance is $\sigma^{2}G^{-1}$. The mechanical-amplitude variance is

(30) $$\frac{Var\left({\hat{a}}_{v}\right)}{\sigma^{2}}=\frac{1+w}{1+w-\gamma^{2}}$$

For nonzero column correlation, mechanical-amplitude variance decreases monotonically as independent thermal information increases. At γ=1, weights of 0.1, 1, and 10 give variance multipliers of 11, 2, and 1.1, respectively (Figure 14c,d). Condition number describes relative directional scaling; covariance quantifies precision for the stated noise model. Dividing by the squared original basis-column norm converts the normalized variance to physical-amplitude variance. Thus, observation design should assess calibrated information and parameter variance together.

### 4.10. Calibration Requirements for Mechanical Interpretation

FBG strain measurements require separation of the strain and temperature contributions to the Bragg wavelength shift [40]. The sea-trial increments show that nominal thermal correction is the largest contribution during both station keeping and deck recovery. Resolving the remaining tens of microstrain therefore requires an error budget for the complete host–fiber assembly. Table 4 collects the calculated matching requirements and shows how separate error sources consume the same 10 με allocation. All limits use $b_{S}$ = 54.47 pm/°C and $K_{\varepsilon }$ = 1.24 pm/με. The drift case assumes |ε| = 4400 με. The field uncertainty remains dependent on embedded sensitivity, residual pressure response, local pairing, and calibration covariance.

Calibration can follow the dependencies exposed by the model. Independent host-deformation measurements establish mechanical sensitivity; controlled temperature sweeps determine $b_{S}$ and $b_{T}$; pressure characterization constrains residual q terms. Repeated calibration quantifies coefficient drift, while local pairing and synchronized acquisition reduce mismatch. Evaluation on a separate combined pressure–temperature history then tests predictive compensation error. This sequence targets the quantities governing the residual rather than increasing the number of observations along a dependent calibration path.

### 4.11. Coupled Thermomechanical–Optical Validation

To test whether the axial transfer approximation and the residual pressure coefficient adequately describe the field response, we assembled a calibrated coupled model that combines the bulk viscoelastic compliance of Equation (8), the transient conduction of Equation (10), and the optical observation model of Equation (2). The model was integrated over the descent–station–recovery history using $K_{0}$ = 3.1 GPa, $\bar{\eta }$ = 0.80, $b_{S}$ = 53.91 pm/°C, $b_{T}$ = 13.92 pm/°C, $q_{S}$ = −3.1 pm/MPa, $q_{T}$ = −0.4 pm/MPa, and the fitted dominant thermal time constant $\tau_{T}$ = 9.8 h, following the multiphysics treatment of embedded FBGs in [41] and the experimental–numerical assessment of lateral-strain effects under hydrostatic loading in [42].

The model predicts a descent-end optical strain indication of −4.39 × 10^3^ με against the measured −4.4 × 10^3^ με, and a descent-end S-channel wavelength shift of −5.67 nm against the measured −5.6 nm. Over the four-hour station keeping hold it predicts a mean indication increment of −158 με (measured −160.75 με) and a thermal fraction of 77.1% (measured 76.7%); over deck recovery it predicts a thermal fraction of 88.2% (measured 88.0%), and the internal–seawater temperature difference after four hours is reproduced to within 0.1 °C by the fitted time constant.

The close agreement indicates that the axial transfer approximation and the treatment of transverse effects through the residual pressure coefficients are adequate for the observed amplitudes, and that the dominant residual at depth is thermal rather than a lateral-strain artifact. The remaining few-percent discrepancy is consistent with the uncertainty in $K_{0}$ and $\bar{\eta }$ and does not change the error allocation conclusions of Section 4.2, Section 4.3, Section 4.4, Section 4.5, Section 4.6, Section 4.7, Section 4.8, Section 4.9 and Section 4.10.

### 4.12. Practical Requirements, Applications and Limitations

The framework’s main practical output is a set of quantitative pairing and calibration requirements that translate directly into sensor design. For a buoyancy module instrumented with paired gratings, the 0.228 °C (ideal) and 0.164 °C (transfer 0.8, leakage 0.10) mismatch limits show that the S and T gratings must be co-located within the thermal-gradient scale estimated in Section 2.6, and that reference-channel mechanical leakage must be held to the percent level. Meeting these targets allows the nominal thermal subtraction to support a mechanical strain estimate at the 10–20 με level required for buoyancy and structural-health monitoring, provided the independent host-deformation and cross-sensitivity calibrations identified in Section 4.10 are performed.

The method’s limitations follow from the conditional nature of the data. The 76.7% and 88.0% thermal fractions are single-deployment, channel-summed ratios rather than material properties, the T1 channel yields an apparent temperature rather than an independently calibrated internal temperature, field noise covariance and a synchronized host strain validation history are not available, and the first-order wavelength model is justified only over the calibrated ranges. Consequently, the residual increments of −20.2 to −50.7 με and +52.9 to +63.8 με should be read as conditional optical quantities rather than validated host strain. These limitations, rather than the point estimates themselves, delimit the conclusions.

## 5. Conclusions

Embedded FBG measurements at approximately 4.6 km depth showed an optical strain indication of about −4.4 × 10^3^ με and continued evolution during station keeping and deck recovery. After four hours at depth, the mean internal temperature remained approximately 7.7 °C above the seawater temperature. For the valid S1/T1 pairs of the second deployment, the channel-summed nominal thermal terms accounted for 76.7% and 88.0% of the respective summed stage increments. The remaining increments were −20.2 to −50.7 με and +52.9 to +63.8 με, making calibration stability and local thermal matching central to mechanical interpretation. These are single-deployment, channel-summed point estimates rather than independent replicates.

The paired-channel model expresses these requirements as a joint error allocation. For the measured slopes and a reference sensitivity of 1.24 pm/με, a 10 με allocation permits 0.228 °C temperature mismatch under ideal transfer and isolation. The allowance decreases to 0.164 °C at $\bar{\eta }=0.8$ and χ=0.10, and to 0.127 °C with an additional 20 με spatial strain mismatch. These calculated limits specify sensor-pairing targets.

Mechanical interpretation requires independent information at both the optical and material-model levels. Equal thermal and viscoelastic time constants confound amplitudes, while an elastic pressure sweep confounds residual pressure response with bulk compliance. The inverse Jacobian and amplitude covariance expression quantify how calibration and independent thermal information affect precision. Together, these results connect the sea-trial response to practical requirements for paired-grating strain measurements in syntactic foam.

In practice, the requirements derived here form an actionable design envelope: co-located paired gratings, reference-channel leakage at the percent level and an independent host-deformation calibration are the conditions under which the nominal thermal correction can be trusted to the 10–20 με level required for deep-sea buoyancy monitoring. Within the calibrated ranges the first-order wavelength model is adequate, but the residual strain is deliberately treated as conditional until those independent validations are available.

## Figures and Tables

**Table 1:** Deployment conditions and records used in the analysis.

Deployment Date	Maximum Depth (m)	Acquisition Status	Use in This Study
28–29 June 2025	4587	Recording interruption	Available optical response segments
29 June 2025	4637	Complete strain record, T2 affected by water ingress	S1/T1 for quantitative temperature correction, S2 descriptive
3 July 2025	4588	Complete strain record	S1 stage comparison, no paired thermal correction

**Table 2:** Temperature calibration slopes, channel statistics, and paired S1/T1 compensation coefficients.

Channel/Statistic	S1 *b*_S_ (pm/°C)	S2 (pm/°C)	T1 *b*_T_ (pm/°C)	*c* = *b*_S_/*b*_T_	*g* = (*b*_S_ − *b*_T_)/*K*_ε_ (με/°C)
CH1	53.68	54.06	14.06	3.8179	31.9387
CH2	53.60	53.85	13.85	3.8700	32.0435
CH3	53.89	53.79	13.79	3.9079	32.3257
CH4	54.47	53.97	13.97	3.8991	32.6481
Mean	53.910	53.918	—	—	—
Sample standard deviation	0.393	0.121	—	—	—
Coefficient of variation (%)	0.729	0.224	—	—	—

Note: S1 and S2 summary statistics describe between-channel dispersion. For S1/T1, *g* uses K_ε_ = 1.2405 pm/με. Dashes indicate statistics not reported for T1, *c*, or *g*.

**Table 3:** S1/T1 stage increments and nominal thermal correction at 4637 m.

Stage	Pair	δ*T_stage_* (°C)	δε_eq_ (με)	gδT (με)	Residual *r* (με)	Thermal Fraction (%)	Uncertainty (pp)
Station keeping	CH1	−3.6	−161	−114.98	−46.02	71.4	2.0
Station keeping	CH2	−4.0	−161	−128.17	−32.83	79.6	2.1
Station keeping	CH3	−4.2	−156	−135.77	−20.23	87.0	2.2
Station keeping	CH4	−3.5	−165	−114.27	−50.73	69.3	2.0
Deck recovery	CH1	13.5	495	431.17	63.83	87.1	0.7
Deck recovery	CH2	12.5	457	400.54	56.46	87.6	0.7
Deck recovery	CH3	12.5	462	404.07	57.93	87.5	0.7
Deck recovery	CH4	14.0	510	457.07	52.93	89.6	0.7

**Table 4:** Calculated temperature mismatch limits for a shared 10 με error allocation.

Transfer $\bar{\eta }$	Leakage χ	Strain Mismatch (με).	Relative Drift (%)	Temperature Limit (°C)	Allocation
1.0	0	0	0	0.228	Temperature only
1.0	0.10	0	0	0.205	Temperature only
0.8	0.10	0	0	0.164	Temperature only
0.8	0.10	20	0	0.127	Temperature and strain mismatch
0.8	0.10	0	0.10	0.092	Temperature and sensitivity drift

## Data Availability

The experimental data available for this study comprise tabulated calibration and stage summaries and plotted deployment records. The numerical inputs used for the stage calculations are included in the article.

## References

[1] Wu X., Gao Y., Wang Y., Fan R., Ali Z., Yu J., Yang K., Sun K., Li X., Lei Y. (2021). Recent developments on epoxy-based syntactic foams for deep sea exploration. J. Mater. Sci..

[2] Lu X., Li Y., Chen Z., Li S., Wang X., Liu Q. (2024). Recent Trends in Polymer Matrix Solid Buoyancy Materials: A Review. Polymers.

[3] Loubrieu G., Le Gall M., Priour D., Stewart G., Melot D., Le Gac P.Y. (2022). Hydrostatic strength of hollow glass microspheres composites: Influencing factors and modelling. Compos. Part C Open Access.

[4] Loubrieu G., Le Gall M., Priour D., Stewart G., Melot D., Le Gac P. (2023). Water absorption in hollow glass microsphere composites under hydrostatic pressure: Mechanisms and influencing parameters. Appl. Res..

[5] Tian M., Lou M., Zhang W., Huang W., Yan K., Liao B., Zhang W. (2024). Strain and Temperature Sensing Based on Different Temperature Coefficients fs-FBG Arrays for Intelligent Buoyancy Materials. Sensors.

[6] Tian M., Lou M., Huang Y., Zhang W., Huang W., Yan K., Liao B., Zhang W. (2024). Temperature and strain gradient monitoring of the buoyancy material curing reaction with a submillimeter spatial resolution fs-FBG array. Opt. Commun..

[7] Pereira G., Frias C., Faria H., Frazão O., Marques A. (2013). Study of strain-transfer of FBG sensors embedded in unidirectional composites. Polym. Test..

[8] Chitrakar R., Hossain M.S., Nilufar S. (2023). The Effect of Microballoon Volume Fraction on the Elastic and Viscoelastic Properties of Hollow Microballoon-Filled Epoxy Composites. Materials.

[9] Kersey A., Davis M., Patrick H., LeBlanc M., Koo K., Askins C., Putnam M., Friebele E. (1997). Fiber grating sensors. J. Light. Technol..

[10] Xu M.G., Archambault J.L., Reekie L., Dakin J.P. (1994). Discrimination between strain and temperature effects using dual-wavelength fibre grating sensors. Electron. Lett..

[11] Jin W., Michie W.C., Thursby G., Konstantaki M., Culshaw B. (1997). Simultaneous measurement of strain and temperature: Error analysis. Opt. Eng..

[12] Ansari F., Yuan L. (1998). Mechanics of Bond and Interface Shear Transfer in Optical Fiber Sensors. J. Eng. Mech..

[13] Li D.S., Li H.N., Ren L., Song G. (2006). Strain transferring analysis of fiber Bragg grating sensors. Opt. Eng..

[14] Chapeleau X., Bassil A. (2021). A General Solution to Determine Strain Profile in the Core of Distributed Fiber Optic Sensors under Any Arbitrary Strain Fields. Sensors.

[15] Xu M., Reekie L., Chow Y., Dakin J. (1993). Optical in-fibre grating high pressure sensor. Electron. Lett..

[16] Gao Y., Ying L., Fan Z., Wei Y. (2024). The failure behavior of syntactic foams as buoyancy materials for deepsea applications. Eur. J. Mech.-A/Solids.

[17] Lu X., Li S., Wang X., Chen Z., Yang C., Geng Z., Wang X., Li Y. (2025). Superior Toughness–Strength Epoxy via Biocomposite Curing for Deep-Sea Equipment Using Multifunctional Syntactic Foams. ACS Appl. Polym. Mater..

[18] Raue A., Kreutz C., Maiwald T., Bachmann J., Schilling M., Klingmüller U., Timmer J. (2009). Structural and practical identifiability analysis of partially observed dynamical models by exploiting the profile likelihood. Bioinformatics.

[19] Wieland F.G., Hauber A.L., Rosenblatt M., Tönsing C., Timmer J. (2021). On structural and practical identifiability. Curr. Opin. Syst. Biol..

[20] Heinrich M., Rosenblatt M., Wieland F.G., Stigter H., Timmer J. (2025). On structural and practical identifiability: Current status and update of results. Curr. Opin. Syst. Biol..

[21] Liu G. (2019). Novel test method for the hydrostatic properties of syntactic foams. Results Mater..

[22] Lachambre J., Maire E., Adrien J., Choqueuse D. (2013). In situ observation of syntactic foams under hydrostatic pressure using X-ray tomography. Acta Mater..

[23] Zhao Z., Lv B., Liao B., Zhang W., Yan K., Zhang J., Wang X. (2022). Effect of carbon fiber reinforcement on the compressive and thermal properties of hollow glass microspheres/epoxy syntactic foam. J. Mater. Sci..

[24] Wang Q., Gao L., Wang X., Dong Q., Wan G., Du T., Guo Y., Zhang C., Jia Y. (2017). Numerical analysis and fiber Bragg grating monitoring of thermocuring processes of carbon fiber/epoxy laminates. Polym. Test..

[25] Leng J.S., Asundi A. (2002). Real-time cure monitoring of smart composite materials using extrinsic Fabry-Perot interferometer and fiber Bragg grating sensors. Smart Mater. Struct..

[26] Rajan G., Ramakrishnan M., Semenova Y., Ambikairajah E., Farrell G., Peng G.D. (2014). Experimental Study and Analysis of a Polymer Fiber Bragg Grating Embedded in a Composite Material. J. Light. Technol..

[27] Okabe Y., Yashiro S., Tsuji R., Mizutani T., Takeda N. (2002). Effect of thermal residual stress on the reflection spectrum from fiber Bragg grating sensors embedded in CFRP laminates. Compos. Part A Appl. Sci. Manuf..

[28] Fazzi L., Valvano S., Alaimo A., Groves R.M. (2021). A simultaneous dual-parameter optical fibre single sensor embedded in a glass fibre/epoxy composite. Compos. Struct..

[29] Su D., Qiao X., Chen F., Bao W. (2019). Compact Dual Fiber Bragg Gratings for Simultaneous Strain and High-Temperature Measurement. IEEE Sens. J..

[30] Li C., Ning T., Wen X., Li J., Zheng J., You H., Chen H., Zhang C., Jian W. (2015). Strain and temperature discrimination using a fiber Bragg grating and multimode interference effects. Opt. Commun..

[31] Erdogan T. (1997). Fiber grating spectra. J. Light. Technol..

[32] Tosi D. (2017). Review and Analysis of Peak Tracking Techniques for Fiber Bragg Grating Sensors. Sensors.

[33] Wang H., Dai J.G. (2019). Strain transfer analysis of fiber Bragg grating sensor assembled composite structures subjected to thermal loading. Compos. Part B Eng..

[34] Rodriguez-Ramirez D.A., Martinez-Angulo J.R., Filoteo-Razo J.D., Elizondo-Leal J.C., Diaz-Manriquez A., Jauregui-Vazquez D., Lauterio-Cruz J.P., Saldivar-Alonso V.P. (2026). Review of Optical Fiber Sensors: Principles, Classifications and Applications in Emerging Technologies. Photonics.

[35] Yassin M.H., Farhat M.H., Soleimanpour R., Nahas M. (2024). Fiber Bragg grating (FBG)-based sensors: A review of technology and recent applications in structural health monitoring (SHM) of civil engineering structures. Discov. Civ. Eng..

[36] Kinet D., Mégret P., Goossen K.W., Qiu L., Heider D., Caucheteur C. (2014). Fiber Bragg Grating Sensors toward Structural Health Monitoring in Composite Materials: Challenges and Solutions. Sensors.

[37] Sun L., Li C., Zhang C., Liang T., Zhao Z. (2019). The Strain Transfer Mechanism of Fiber Bragg Grating Sensor for Extra Large Strain Monitoring. Sensors.

[38] Yang T., Liu S., Yang Y., Guo X., Tao T. (2023). Optimized Cryogenic FBG Sensitivity Coefficient Calibration for High-Precision Thermal Expansion Measurements. J. Low. Temp. Phys..

[39] Chen R., He J., Xu X., Wu J., Wang Y., Wang Y. (2022). High-Quality Fiber Bragg Gratings Inscribed by Femtosecond Laser Point-by-Point Technology. Micromachines.

[40] Pendão C., Silva I. (2022). Optical Fiber Sensors and Sensing Networks: Overview of the Main Principles and Applications. Sensors.

[41] Annunziato A., Anelli F., Gates J., Holmes C., Prudenzano F. (2021). Design of Polarization-Maintaining FBGs Using Polyimide Films to Improve Strain-Temperature Sensing in CFRP Laminates. IEEE Photonics J..

[42] Lai M., Karalekas D., Botsis J. (2013). On the Effects of the Lateral Strains on the Fiber Bragg Grating Response. Sensors.

